# Presynaptic dysfunction in *CASK*-related neurodevelopmental disorders

**DOI:** 10.1038/s41398-020-00994-0

**Published:** 2020-09-14

**Authors:** Martin Becker, Francesca Mastropasqua, Jan Philipp Reising, Simon Maier, Mai-Lan Ho, Ielyzaveta Rabkina, Danyang Li, Janina Neufeld, Lea Ballenberger, Lynnea Myers, Viveka Moritz, Malin Kele, Josephine Wincent, Charlotte Willfors, Rouslan Sitnikov, Eric Herlenius, Britt-Marie Anderlid, Anna Falk, Sven Bölte, Kristiina Tammimies

**Affiliations:** 1 Center of Neurodevelopmental Disorders (KIND), Division of Neuropsychiatry, Department of Women’s and Children’s Health, Karolinska Institutet, and Center for Psychiatry Research, Region Stockholm, Sweden; 2 Astrid Lindgren Children’s Hospital, Karolinska University Hospital, Region Stockholm, Sweden; 3 Department of Women’s and Children’s Health, Karolinska Institutet, Stockholm, Sweden; 4 Department of Psychiatry and Psychotherapy, Medical Center—University of Freiburg, Faculty of Medicine, University of Freiburg, Freiburg im Breisgau, Germany; 5 Department of Radiology, Nationwide Children’s Hospital, Columbus, OH USA; 6 Interdisciplinary Center for Neurosciences, Heidelberg University, Heidelberg, Germany; 7 Gustavus Adolphus College, St. Peter, Minnesota USA; 8 Department of Neuroscience, Karolinska Institutet, Stockholm, Sweden; 9 Department of Molecular Medicine and Surgery, Karolinska Institutet, Stockholm, Sweden; 10 Department for Clinical Genetics, Karolinska University Hospital, Stockholm, Sweden; 11 Department of Neuroradiology, Karolinska University Hospital and Department of Clinical Neuroscience, Karolinska Institutet, Stockholm, Sweden; 12 Child and Adolescent Psychiatry, Stockholm Health Care Services, Stockholm County Council, Stockholm, Sweden; 13 Curtin Autism Research Group, School of Occupational Therapy, Social Work and Speech Pathology, Curtin University, Perth, Western Australia

**Keywords:** Personalized medicine, Molecular neuroscience

## Abstract

*CASK*-related disorders are genetically defined neurodevelopmental syndromes. There is limited information about the effects of *CASK* mutations in human neurons. Therefore, we sought to delineate *CASK-*mutation consequences and neuronal effects using induced pluripotent stem cell-derived neurons from two mutation carriers. One male case with autism spectrum disorder carried a novel splice-site mutation and a female case with intellectual disability carried an intragenic tandem duplication. We show reduction of CASK protein in maturing neurons from the mutation carriers, which leads to significant downregulation of genes involved in presynaptic development and of CASK protein interactors. Furthermore, *CASK*-deficient neurons showed decreased inhibitory presynapse size as indicated by VGAT staining, which may alter the excitatory–inhibitory (E/I) balance in developing neural circuitries. Using in vivo magnetic resonance spectroscopy quantification of GABA in the male mutation carrier, we further highlight the possibility to validate in vitro cellular data in the brain. Our data show that future pharmacological and clinical studies on targeting presynapses and E/I imbalance could lead to specific treatments for *CASK*-related disorders.

## Introduction

The identification of genetic variants underlying neurodevelopmental disorders (NDDs), such as intellectual disability (ID), autism spectrum disorder (ASD), and attention-deficit hyperactivity disorder (ADHD), as well as epilepsies, has increased at a rapid pace in recent years with a high level of pleiotropy across the conditions^1–3^. Findings indicate shared molecular mechanisms underlying the diverse clinical phenotypes. However, for many of the identified risk variants and genes, the molecular and neuronal outcomes are not well understood. One pleiotropic gene is the *calcium/calmodulin-dependent serine protein kinase* (*CASK*), located on chromosome Xp11.4, in which pathogenic variants underlie a range of NDDs. Genetic variants in *CASK* were first described in cases with microcephaly with pontine and cerebellar hypoplasia (MICPCH), followed by the identification in cases with X-linked ID (XL-ID), developmental delay (DD), and ASD^1,4–6^. The majority of cases reported with *CASK*-related disorders are females with MICPCH, caused by heterozygous loss-of-function (LoF) variants^5,7^. Skewed X-chromosome inactivation (XCI) has shown protective effects against more severe phenotypes^5,8^. Missense variants, found both in females and males, cause microcephaly, XL-ID, DD, or ASD^1,4,6,8–10^. The molecular pathways and cellular phenotypes associated with genetic variants causing *CASK*-related disorders are mostly unknown, especially in human neurons.

CASK is ubiquitously expressed with high expression in the developing human brain^11^. The protein structure consists of scaffolding domains L27, PDZ, and SH3, as well as a Ca^2+^/calmodulin-dependent protein kinase and guanylate kinase domain^12^. In neurons, CASK is involved in pre- and postsynaptic signaling. At the presynapse, CASK regulates the synaptic vesicle exocytosis and neuronal cell adhesion through a tripartite complex with VELI1 (*LIN7A)* and MINT1 (*APBA1)* and direct interaction with NRXN1 (*NRXN1*)^13,14^. The tripartite protein complex VELI1–MINT1–CASK is unaffected by XL-ID and MICPCH missense mutations^9^. Instead, the CASK–NRXN1 interaction is disrupted, suggesting that the absence of this interaction is associated with the MICPCH phenotype. In the postsynaptic density, CASK contributes to the regulation of ionotropic receptor trafficking^15^. Electrophysiological assessment of CASK-negative neurons from mice showed a shift in excitatory–inhibitory (E/I) balance with increased spontaneous miniature excitatory postsynaptic currents (mEPSC) and decreased miniature inhibitory postsynaptic currents (mIPSC)^16,17^. Interestingly, *Nrxn1* KO mouse neurons and human neuronal models with *NRXN1* LoF mutations have elevated *CASK* levels and decreased mEPSCs^18^. In addition to the role at the synapse, CASK has been shown to act as a co-regulator of transcription through TBR1 (*TBR1*), CINAP (*TSPYL2*), and BCL11A (*BCL11A*)^12,19,20^, all of which have been implicated in NDDs^21–23^.

Here, we elucidate the consequences of *CASK* pathogenic variants using human induced pluripotent stem cell (iPSC)-derived neuronal models from two mutation carriers, one diagnosed with ASD and one with MICPCH. Our transcriptional, morphological, and functional analyses reveal that reduced levels of CASK are sufficient to induce aberrant presynaptic development, and have wide effects on its interaction network, as well as on multiple developmental pathways. In addition, for the first time, we explore the possibility to validate neuronal findings from iPSC models using in vivo brain data. In conclusion, our data suggest that *CASK*-related disorders are synaptopathies that affect the E/I balance in maturing neural circuits.

## Materials and methods

### Identification of mutation carriers

The mutation carriers were recruited to the study from The Roots of Autism and ADHD study in Sweden (RATSS) and Clinical Genetics at the Karolinska University Hospital. Comprehensive phenotyping of the ASD case and his cotwin has been described elsewhere^24,25^. Phenotyping, genotyping, and clinical assessment of the girl with MICPCH was described earlier^26^. Written informed consent was obtained from affected individuals and their parents prior to the study. The study was approved by the regional and national ethical boards in Sweden, and it has been conducted in accordance with the Declaration of Helsinki for medical research involving human subjects, including research on identifiable human material and data.

### Whole-exome sequencing and data processing

For the identification of the genetic alterations, we performed microarray analysis (for both cases) and whole-exome sequencing (for the twin pair and their parents). For de novo variant detection, the parents were included in the genetic analyses. DNA was extracted from whole blood and saliva samples using standard methods. The detection of CNVs has been described earlier^26,27^. Identification of clinically relevant variants from WES is detailed in the Supplementary material and methods. Categorization was made based on the criteria from the American College of Medical Genetics and Genomics (ACMG)^28^.

### Cell culture of iPS and NES cells

Human iPSC cells were derived for the *CASK-*mutation carriers diagnosed with MICPCH and ASD, using a previously described procedure^29^. A summary of the protocol and quality control of the iPS cells is described in the Supplementary materials and methods. Dual-SMAD inhibition was applied to derive NES cells from human iPS cells as described previously^30,31^.

NES cells were seeded on plastic surface, precoated with 20 µg/ml poly-ornithine (Sigma Aldrich) and 1 µg/ml laminin (Sigma Aldrich) in DMEM/F12 + glutamax medium (Gibco) supplemented with 0.5× B-27 (Gibco), 1× N-2 (Gibco), and 10 U/ml penicillin/streptomycin (Gibco). Cells were maintained in a 5% CO_2_ atmosphere at 37 °C. Two-third of the medium was changed every second day of differentiation with 0.4 µg/ml laminin added. Neurons were differentiated for 8, 16, or 28 days. Cells harvested for time point day 0 were cultured for 2 days in NES cell medium.

### RNA extraction and RT-qPCR

Cells were lysed in TRIzol reagent (Invitrogen), and RNA was isolated using the ReliaPrep RNA Cell Miniprep (Promega #A1222). The RNA was reverse-transcribed using iScript cDNA Synthesis Kit (BioRad) and cDNA quantified with SsoAdvanced Universal SYBR Green Supermix (BioRad) following the manufacturer’s protocols on a CFX96 thermal cycler (BioRad). CFX Manager software was used to record amplification curves and to determine Ct values. RT-qPCR reactions were performed in technical triplicates. We calculated the ΔCt to the GAPDH housekeeping gene and ^ΔΔ^Ct to control cell lines. We used three biological replicates of cells seeded at different passages, if not stated otherwise in the figure legends. Statistical significance between cell lines was determined with ANOVA and post hoc Tukey HSD in R.

### Protein quantification using capillary western blot

Cells were dissociated in extraction buffer (50 mM Tris-HCl, 100 mM NaCl, 5 mM EDTA, and 1 mM EGTA) using plastic cell scraper and lysed with 6 short sonication bursts at 36% amplitude (Vibra-Cell VCX-600, Sonics). About 250 µg/ml protein was loaded for Simple-Western WES (ProteinSimple) quantification with antibodies for CASK (1:500 Novus Biologicals NBP2-41181), beta-actin (1:100 Abcam ab8227), and GAPDH (1:5000 Sigma G9545). Compass Software For Simple Western (Version 4.0.0) was used to identify protein-specific peaks. In the chromatogram, the peak area was used for protein quantification. We normalized CASK protein for housekeeping protein. For time point days 0, 8, and 16, we obtained two and three biological replicates for time point 28 and siRNA-mediated knockdown of CASK. Biological replicates were seeded at different dates, using different passages. Statistical significance between cell lines was determined with ANOVA and post hoc Tukey HSD in R.

### Immunofluorescence

Cells were cultured on glass coverslips for the indicated differentiation time and fixed for 20 min in 4% formaldehyde. Primary antibodies used are CASK-NBP2-41181, 1:500 (Novus bio), MAP2-M2320, 1:500 (Sigma Aldrich), VGLUT1-135304, 1:250 (Synaptic systems), Homer-1-160011, 1:250 (Synaptic systems), VGAT-131003, 1:500 (Synaptic systems), Synapsin-1/2-106006, 1:500 (Synaptic systems), Gephyrin-147021, 1:250 (Synaptic systems), Nestin-MAB5326-KC, 1:1000 (Merck-Millipore), and SOX2-AB5603, 1:1000 (Merck-Millipore). All images were taken with LSM 700 Zeiss Confocal Microscope (Zeiss Plan-Apochromat 63×/1.40na Oil DIC Objective M27), with 63× magnification at 1024- × 1024-pixel [pxl] resolution, resulting in an aspect ratio of 0.099233 µm per pixel.

ImageJ Particle Analyzer was used to count CASK puncta and cell nuclei. Quantification of particle number and size was done in R. Four independent replicates, seeded at different dates and passages, were obtained for each cell line. Synaptic marker particle size and number were quantified with the ImageJ plugin Synapse Counter, using default settings^32^. For the Synapsin-1/2–Homer-1 and VGlut–Homer-1 co-staining, we obtained four independent replicates for each cell line. For the VGAT–Homer-1 and VGAT–Gephyrin co-staining, we obtained three independent replicates.

### siRNA-mediated gene knockdown

NES cells were seeded for differentiation and the next day transfected with 0.5 µM Accell SMARTpool siRNA targeting *CASK* mRNA (Dharmacon #E-005311-00-0010) or 0.5 µM Accell Green Nontargeting siRNA (Dharmacon #D-001950-01-20) according to standard protocol. Three biological replicates were harvested for each protein and RNA analysis.

### Single-cell RNA sequencing

Cells were dissociated from culture surface with 2 min of trypsin incubation and subsequent trypsin inhibition. After 3 min centrifugation at 300 rcf, the cells were resuspended in cold Dulbecco’s phosphate-buffered saline, and single cells sorted by size into lysis buffer using BD FACS Aria III. Smart-Seq2 library preparation and sequencing were done with the Eukaryotic Single Cell Genomics (ESCG) facility in the SciLifeLab, Stockholm^33^. In total, 384 sorted wells were sequenced with a total of 209.6 M reads and an average sequence depth of 550,000 reads per cell. The individual counts per gene and cell were reported in a count matrix (GSE140572) and used for further analysis. The scImpute package was used to calculate dropout expression values from the count matrix^34^. Cells with less than 50,000 read counts in less than 2000 genes were removed from analysis. Differential gene expression and cell clustering were done using the Seurat package^35^. To obtain mutant CASK reads, we created a custom genome with STAR that included the cDNA sequence of the CASK gene with the duplicated exons 4 and 5 and realigned all sequence reads. Exon 5–exon 4 spanning sequence reads were analyzed using vcftools.

### Bulk RNA sequencing

We extracted RNA samples of five biological replicates per cell line that were seeded at different dates of different passages. Samples were delivered to NGI Sweden for library preparation and sequencing. We obtained on average 50.4 million reads per sample with a minimum of 96.9% reads aligned to protein-coding regions. Sample read counts are supplied in GSE140572. Replicates of each cell line cluster together with the exception of one MICPCH replicate that was removed from analysis.

Differential gene expression was calculated from gene counts using DESeq2 (v1.24.0) in R. To determine a differential expression for individual cases, we used passage and cell lines as independent variables and compared ASD_CASK_SS_ with male control and MICPCH_CASK_dup4/5_ with female control. Top ranking genes for comparison between ASD_CASK_SS_ and MICPCH_CASK_dup4/5_ were filtered at an adjusted *p* value of 1E−5 (BHA), with more than 20 reads and log2 fold change (LC) greater than 0.5. The Venn diagram was visualized in R using the VennDiagramm package (v1.6.20), and overrepresentation analysis was performed using WebGestaltR (v0.4.2).

Pathway analysis was done according to previously described protocols^36^. In short, the ranked gene expression list was used in Gene set enrichment analysis (GSEA) (Version 3.0)^37^ and enriched categories were visualized in Cytoscape (v3.7.0) with Enrichment Map (v3.1.0) and AutoAnnotate^38,39^. In addition, GSEA was performed on CASK PPI (downloaded from PathwayCommons; PCViz: CASK on 08.Oct.2019), an in-house NDD gene list (Supplementary Material and Methods), and SFARI sublist.

### Deconvolution

The deconvolution of bulk RNASeq data was done using the BSEQ-sc package^40^. BSEQ-sc uses cell-type-specific marker genes from single-cell RNA transcriptomes to predict cell-type proportions underlying bulk RNA transcriptomes. Deconvolution was done on increasing numbers of significant genes, and the predictions were stable when using 10 or 20 most significant marker genes. Statistical differences in the estimated cell proportions between patient and both control cell lines were done using ANOVA and post hoc Tukey HSD test.

### Calcium imaging

Calcium imaging was performed according to protocols described earlier^41^. Cells were loaded with Fluo-8 for 45 min at 37 °C with 5% CO_2_ and imaged for 5 min at 37 °C at normal atmosphere. Mean gray values of ROIs were recorded over the 5-min preprocessed time series to obtain traces of neuronal activity. Traces were resampled to 2 Hz and smoothed using the python scipy.signal package. Peaks were detected using the PeakCaller Matlab software^42^. Biological replicates at 4 weeks: female control (*n* = 2), male control (*n* = 2), ASD_CASK_SS_ (*n* = 1), and MICPCH_CASK_dup4/5_ (*n* = 2). Five weeks: female control (*n* = 2), male control (*n* = 2), ASD_CASK_SS_ (*n* = 5), and MICPCH_CASK_dup4/5_ (*n* = 2). Active cells per cell line were pooled across replicates, and statistical differences at 4 and 5 weeks were calculated using pairwise Wilcoxon Rank Sum test followed with Bonferroni correction.

### [1H]MRS

Proton magnetic resonance spectroscopy ([1H]MRS) data were available from the EU-AIMS Longitudinal European Autism Project (LEAP) autism twin cohort, including ASD_CASK_SS_^43^. MR data were acquired on MR750, 3 Tesla scanner (GE Healthcare, Milwaukie, USA), with 8-channel receiver array coil (in-VIVO Inc.). MRS data were acquired with MEGA-PRESS pulse sequence, te/tr = 68/2000 ms, number of averages 192, phase cycle 8 steps, and bandwidth 5 kHz for three voxels with volumes 19.3 mL (Medial PFC), 19.5 mL (Putamen-GPS), and 13.6 mL (DLPFC). GABA concentration of all three voxels was quantified from the MEGA-PRESS difference spectra using the “Gannet” GABA-MRS analysis toolkit, version 3.0^44^. Metabolite values were scaled to water and expressed in institutional units (IU). Water-scaled GABA concentrations represent GABA as well as related macromolecules, and are therefore referred to as GABA+ concentrations. GABA concentrations were adjusted for the average proportion of partial GM and white matter (WM) volume in each voxel across all subjects. For tissue correction, the GABA concentration ratio α between GM and WM was set to 0.5^45^. All spectra were visually evaluated for the quality of GABA fit. For the final analysis, 45 spectra from the Putamen (20 cotwins to individuals with NDDs), 33 from the MFC (11 cotwins to individuals with NDDs), and 44 from the DLPFC (19 cotwins to individuals with NDDs) of the control subjects could be included.

## Results

### *CASK* variants lead to mutant transcripts and reduced CASK_WT_ expression

**Fig. 1:**
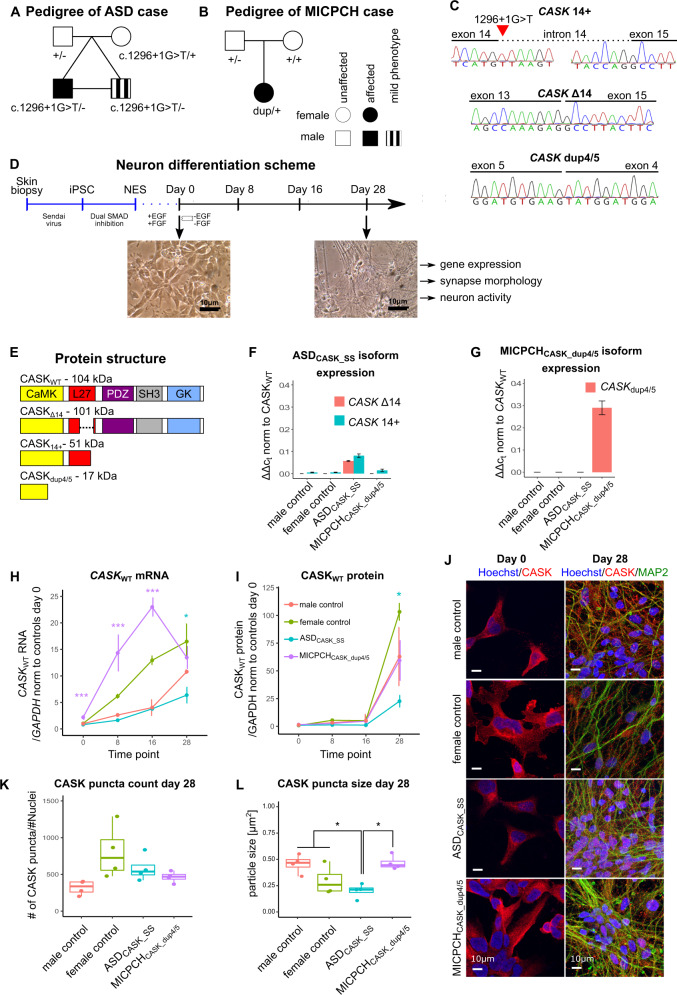
CASK mutations affect wild-type expression in carriers. **a** Pedigree of a male diagnosed with ASD and his monozygotic cotwin who exhibits autistic traits, both carrying a splice-site mutation in the X-linked *CASK* gene (chrX: 41,586,906 C>A (hg38); NM_001126055: c.1296+1 G>T) inherited from a typically developed mother. **b** Pedigree of a female diagnosed with MICPCH, carrying a de novo duplication of 54.9 kb surrounding exons 4 and 5 (chrX:41710324–41765176 (hg38)) of the *CASK* gene. **c** Sanger chromatogram of *CASK* mRNA isoforms in cDNA of ASD_CASK_SS_ fibroblasts and MICPCH_CASK_dup4/5_ NES cells. **d** Schematic summary of the differentiation protocol from skin biopsy to maturing neurons and representative phase-contrast microscopy images of male control NES cells and day 28 neurons. **e** Protein domain structure of CASK_WT_ and in silico-predicted domain structure of CASK_Δ14_, CASK_14+_, and CASK_dup4/5_. **f** RT-qPCR quantification of *CASK*_Δ14_ and *CASK*_14+_ expression in three biological replicates of case and control NES cells. **g** RT-qPCR quantification of *CASK*_dup4/5_ in three biological replicates of case and control NES cells. **h** RT-qPCR quantification of *CASK*_WT_ in three biological replicates of differentiating neurons. **i** Protein quantification of CASK_WT_ protein in two biological replicates of differentiating neurons using capillary western blot quantification. **j** Representative confocal microscopy images of NES cells (day 0) and differentiated neurons (day 28) immunostained for CASK (red), MAP2 (green), and Hoechst (blue). Scale = 10 µm. **k**, **l** Quantification of CASK puncta **k** number and **l** size from confocal images (*n* = 4 per cell line). Statistical differences between cases and controls were calculated using ANOVA with post hoc Tukey HSD. *p < 0.05, ***p < 0.001. Asterisks in **a** and **b** are color-coded according to case cell lines.

To analyze the converging molecular and cellular consequences of pathogenic variants affecting *CASK* across the associated NDD spectrum, we recruited two families with unique *CASK* genetic alterations, representing ASD and MICPCH phenotypes. First, we identified a male individual diagnosed with ASD carrying a *CASK* mutation from the Roots of Autism and ADHD Twin Study in Sweden (RATSS)^24^. He had a primary diagnosis of ASD and general intellectual abilities in the upper average range without cognitive impairments (total IQ = 121) and additional diagnoses of ADHD and tic disorder (Fig. 1a, Supplementary Table 1). His monozygotic cotwin was diagnosed with ASD prior to the RATSS study. However, the cotwin did not fulfill clinical consensus ASD diagnostic criteria at reassessment during the study, but still exhibited autistic symptoms (Supplementary Table 1). Anatomical magnetic resonance imaging (MRI) scans of both twins revealed mild cerebral atrophy and mild cerebellar hypoplasia within normal variation (Supplementary Fig. 1a, b). No clinically significant copy number variants were found in the twin pair. Rare variants from the exome sequencing were prioritized based on variant effect, inheritance mode, and genes involved in NDDs. We identified a novel maternally inherited splice-site variant in intron 14 of the *CASK* gene in both twins (chrX: 41,586,906 C>A (hg38); NM_001126055: c.1296+1 G>T, Fig. 1a, Supplementary Fig. 1c). The variant disrupts the consensus splice donor site at +1 G nucleotide (Supplementary Fig. 1d). *CASK* is intolerant to LoF mutations (pLI = 1), and three putative LoF splice-site mutations reported in the general population are outside the main transcript or tissue-specific transcripts, as reported by the GTEx consortium^46,47^. In addition, both twins carry a de novo heterozygous missense mutation affecting *DIAPH1* exon 15 (chr5: 141,574,906 C>A (hg38); NM_005219: c.1528 G>A: p.E510K, Supplementary Fig. 1e). Heterozygous truncating mutations of this gene are reported in cases of congenital deafness^48^, and homozygous LoF mutations are linked to microcephaly^49^. None of the reported dominant mutations in *DIAPH1* have been linked to NDDs. Of interest, slight hearing problems in one ear were reported for one of the twins (Supplementary Table 1).

We also recruited a female with a classical presentation of MICPCH syndrome, caused by a de novo duplication of 54.9 kb at chromosome Xp11.4, spanning two exons of *CASK*^26^ (Fig. 1b). Her degree of ID is in accordance with earlier-described individuals with MICPCH. She did not communicate verbally, had pontocerebellar hypoplasia, scoliosis, optic nerve hypoplasia, and infantile spasms and epilepsy. Wincent et al. provided detailed description of the individual (referred to as patient 7 in their study), including clinical symptoms, structural brain MRI, and genetic analysis.

To investigate the consequences of the identified splice-site and duplication variants to *CASK* mRNA, we obtained fibroblasts from the two mutation carriers hereafter referred to as ASD_CASK_SS_ and MICPCH_CASK_dup4/5_. The obtained fibroblasts were transformed into iPSCs. The iPSCs had pluripotent marker expression, normal karyotype, and matched donor genotypes (Supplementary Fig. 1f, g). Furthermore, the iPSCs were differentiated to long-term self-renewing neuroepithelial-like stem cells (further referred to as NES cells, Fig. 1d)^31^. NES cell lines derived from two sex-matched typically developed individuals were obtained for controls^29,31^. We detected two mutant *CASK* mRNA isoforms in the NES cells from ASD_CASK_SS_ (Fig. 1c). One isoform lacks exon 14 (CASK_∆14_), and one isoform retains 196 nucleotides of intron 14 (CASK_14+_), resulting in a premature stop codon (Fig. 1e). Although ASD_CASK_SS_ is hemizygous, we detected wild-type *CASK* mRNA (CASK_WT_) with intact joining of exons 14 and 15 constituting the majority of the expression. A minor proportion of *CASK* mRNA consisted of mutant CASK_∆14_ and CASK_14+_ isoforms with approximately 6% and 8%, respectively (Fig. 1f). CASK_14+_ isoform was detected at low levels in control and MICPCH samples, presenting likely pre-mRNA. The presence of CASK_WT_ in the cells of ASD_CASK_SS_ indicates rescue mechanisms for normal splicing possibly through mRNA editing. The MICPCH_CASK_dup4/5_ NES cells expressed an mRNA isoform with a tandem duplication of exons 4 and 5 (Fig. 1c), which leads to a frame shift and early stop codon in the *CASK* coding sequence (Fig. 1e). This mutant isoform constitutes ~29% of total *CASK* mRNA in MICPCH_CASK_dup4/5_ cells (Fig. 1g). In summary, we show that NES cells from both mutation carriers express CASK_WT_ and detectable levels of mutant mRNA isoforms that may interfere with CASK function if translated to protein.

### Reduction of CASK expression in maturing neurons from mutation carriers

Next, we differentiated NES cells to neurons and investigated the neurodevelopmental expression of *CASK* variants in vitro. To cover changes occurring during transition from neuroepithelial stem cells to maturing neurons, we collected RNA and protein from 8, 16, and 28 days of neuronal differentiation (Fig. 1d). None of the predicted mutant protein isoforms were detected from capillary western blotting using the N-terminal antibody (Supplementary Fig. 2a), suggesting that mutant mRNAs are removed by nonsense-mediated decay (NMD). *CASK* mRNA and protein expression increased with differentiation in all cell lines (Fig. 1h, i). However, at 28 days of differentiation, the ASD_CASK_SS_ cells expressed significantly reduced levels of CASK mRNA and protein in comparison to both controls (*p* = 0.02 and *p* = 0.01, respectively, ANOVA post hoc Tukey). mRNA but not protein levels were increased in the MICPCH_CASK_dup4/5_ from NES stage to day 16 and leveled with controls on day 28 (day 0 *p* < 0.001, day 8 *p* < 0.001, day 16 *p* = 0.001, and day 28 *p* = 1, ANOVA post hoc Tukey). The protein levels at day 28 are reduced in comparison with the sex-matched female control (*p* = 0.04, ANOVA post hoc Tukey, Supplementary Fig. 2b). In addition to NMD, biallelic expression from both X chromosomes may explain the discrepancy between increased CASK mRNA and normal protein levels in MICPCH_CASK_dup4/5_.

We further investigated the cellular localization of CASK protein in the ASD_CASK_SS_ and MICPCH_CASK_dup4/5_ cells using the N-terminal binding antibody. In NES cells, we observed small CASK puncta in the cytoplasm of all cell lines with no aberrant nuclear staining (Fig. 1j, day 0). Spatial localization of CASK protein as puncta in neurons has been described earlier^50^. With continuing differentiation, the CASK puncta remained in the cytoplasm, including the neurites (Fig. 1j, day 28) with comparable localization in all cell lines. In MICPCH_CASK_dup4/5_, CASK staining was detected in all cells demonstrating that the *CASK*_WT_ allele is active in all cells. Quantification of CASK puncta showed similar numbers per nuclei (*p* = 0.99 ASD_CASK_SS_ vs. controls, *p* = 0.83 MICPCH_CASK_dup4/5_ vs. controls, ANOVA post hoc Tukey, Fig. 1k) with puncta significantly smaller in differentiated ASD_CASK_SS_ neurons compared to controls and MICPCH_CASK_dup4/5_ (*p* = 0.049 and *p* = 0.011, respectively, ANOVA post hoc Tukey, Fig. 1l). We did not observe significant differences in MICPCH_CASK_dup4/5_ neurons compared to controls.

### X-chromosome activity in MICPCH_*CASK_dup4/5*_ cells

**Fig. 2:**
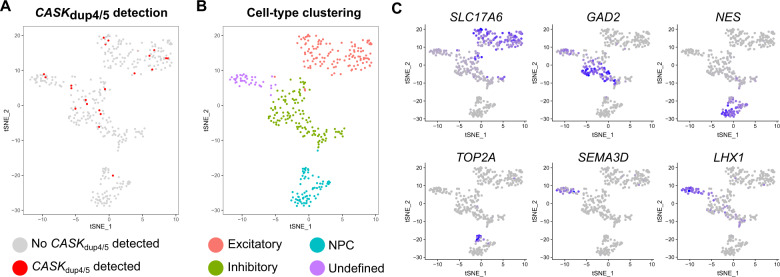
Single-cell transcriptomics of MICPCH_CASK_dup4/5_. **a**–**c** *t*-Distributed stochastic neighbor embedding (tSNE) plot of 383 MICPCH_CASK_dup4/5_ cell transcriptomes after 28 days of differentiation colored by **a** the detection of mutant *CASK*_dup4/5_ expression, **b** the identified cell-type clusters, and **c** expression of *SLC17A6*, *GAD2*, *NES*, *TOP2A*, *SEMA3D*, and *LHX1*.

Random XCI was tested in blood from the MICPCH_CASK_dup4/5_ case in the clinical setting showing a relation between 24% and 76%, consistent with random inactivation. We hypothesized that the discrepancy between CASK mRNA and protein expression during differentiation was due to biallelic expression from wild-type and mutant alleles. To investigate XCI and escape of *CASK* mRNA in our model, we performed SMART-Seq2 single-cell RNA sequencing on MICPCH_CASK_dup4/5_ neurons differentiated for 28 days. We obtained quality reads for 383 cells with a total of 209.6 M reads and an average sequence depth of 550,000 reads per cell. Approximately, 80% of uniquely aligned reads aligned to the genome, 40% to exons, and 30% to introns (Supplementary Fig. 3a, b). We identified the CASK_dup4/5_ specific exon 5–exon 4 junction in 22 cells (Fig. 2a, Supplementary Fig. 3c). We compared this detection rate to the detection of the adjacent exons 4 (62 cells) and 5 (51 cells), indicating that 35–43% of cells expressed CASK_dup4/5_. The frequency of CASK_dup4/5_-expressing cells is indicative of random XCI and comparable to detection levels of the mutant isoform in RT-qPCR (Fig. 1g). The presence of wild-type protein in all MICPCH_CASK_dup4/5_ cells hints toward the escape of *CASK*_WT_ from XCI. As cases and animal models of MICPCH do not show escape of *CASK*_*WT*_ in primary tissue samples, XCI escape is likely an artifact and the phenotype of our cell model milder than that of the patient.

Moreover, we identified cell types in the differentiated neuronal culture from the single-cell transcriptomes and mapped *CASK*_dup4/5_ expression. The cultured cells clustered into four neuronal subtypes, which we identified as neural progenitor cells (NPC), excitatory and inhibitory neurons, as well as one cluster of undefined maturing neurons (Fig. 2b). Excitatory neurons express the *SLC17A6* gene encoding for the vesicular glutamate transporter VGLUT2, and inhibitory neurons express the *GAD2* gene encoding the GAD65 enzyme required for GABA production (Fig. 2c). NPCs express neuronal progenitor marker Nestin (*NES*) and cell-cycle gene *TOP2A*. Selective markers of the fourth cluster included genes involved in axon guidance *SEMA3D* and *LHX1*. As expected from the increase in *CASK* expression during differentiation, we observed higher expression in differentiating neurons compared to NPCs (adj.p = 6E−6, DESeq2, Supplementary Fig. 3d). Expression of the mutant RNA isoform does not cluster within one cell type (Fig. 2a), indicating that CASK protein expression changes are not cell-type specific.

### Reduced CASK levels alter presynaptic development

We performed bulk RNA sequencing of day 28 maturing neurons from all cell lines to identify *CASK*-dependent expression changes. The RNA-sequencing data showed a similar distribution of *CASK* expression for ASD_CASK_SS_ and MICPCH_CASKdup4/5_ as earlier detected by RT-qPCR (Supplementary Fig. 4a). We also quantified the isoform-specific reads from exon–exon spanning fragments (Supplementary Table 2). Similar to RT-qPCR results at NES stage, we detected a low abundance of *CASK-*mutant isoforms *CASK*_14+_ and *CASK*_∆14_ of 4% and 8% in ASD_CASK_SS_, respectively. In addition, we detected both the mutant and reference nucleotide in *CASK*_14+_ reads that span the mutation site (Supplementary Fig. 4b). The de novo variant in *DIAPH1* was present in around 50% of *DIAPH1* transcripts (Supplementary Fig. 4c). In the MICPCH_CASK_dup4/5_ transcriptome, we detected 14% *CASK*_dup4/5_-specific sequence reads aligning to the exon 5–exon 4 junction. MICPCH_CASK_dup4/5_ cells also showed downregulation of the XCI- regulating transcripts *XIST* and *TSIX*, which could explain activation of biallelic *CASK* expression (Supplementary Fig. 4d, e). In line with reduced expression of XCI transcripts, we observed an overall increase in X-chromosome gene expression in the MICPCH_CASK_dup4/5_ cell line that was not present in ASD_CASK_SS_ (Supplementary Fig. 4f, g). Principal component analysis (PCA) separated the mutation carrier cell lines from controls in the first component (Supplementary Fig. 4h), indicating that expression variability between cell lines is mostly explained by the *CASK*-mutation status. The second component of the PCA separated samples by sex.

**Fig. 3:**
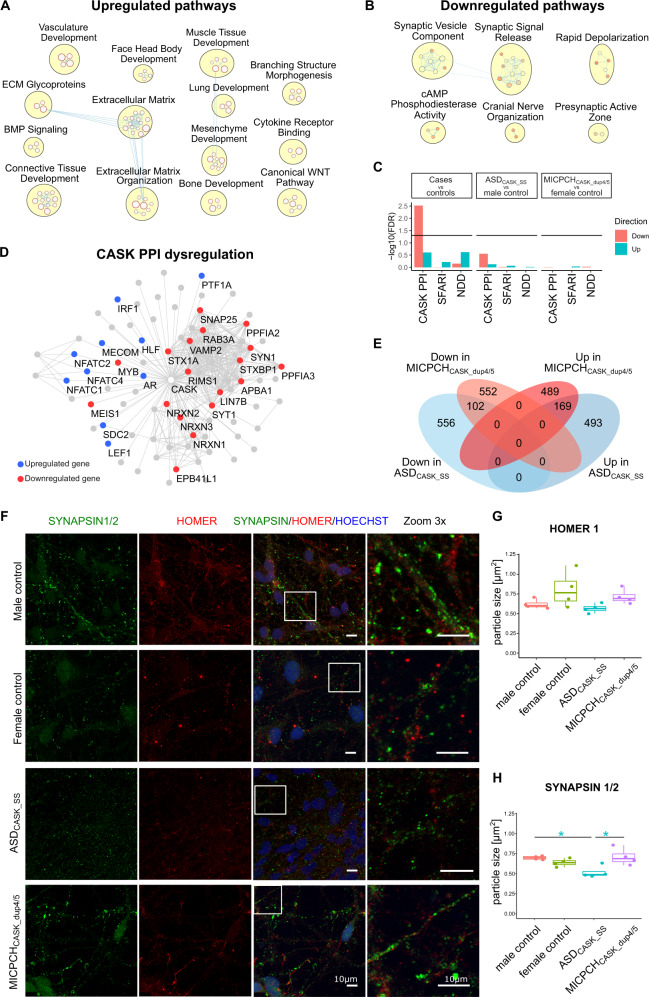
Consistent dysregulation of presynaptic and CASK-interacting genes in bulk RNA sequencing after 28 days of differentiation. **a** Upregulated and **b** downregulated gene sets emerging from GSEA in bulk RNA-sequencing data from mutation carriers compared with controls. **c** GSEA for CASK-interacting proteins (CASK PPI), ASD risk genes (SFARI), and NDD genes (NDD) in bulk RNA-sequencing data from mutation carriers compared with controls. **d** Network of CASK-interacting proteins with upregulation marked in blue and downregulation in red. **e** Venn diagram illustrating the top upregulated and downregulated genes in ASD_CASK_SS_ and MICPCH_CASK_dup4/5_ in comparison with sex-matched controls. **f** Representative confocal microscopy images of neurons differentiated for 28 days and immunostained for Synapsin-1/2 (green), Homer-1 (red), and Hoechst (blue). Scale = 10 µm. **g** Quantification of Homer-1 and **h** Synapsin-1/2 particle size (*n* = 4 per cell line). Statistical differences between cell lines were calculated using ANOVA with post hoc Tukey HSD. *p < 0.05, **p < 0.01, ***p < 0.001. Asterisks are color-coded according to case cell lines.

With support from the PCA results, we performed a pooled analysis of mutation carrier transcriptomes to detect consistent CASK-related expression changes. We detected 4098 differentially (BHA) and a 0.5 log2 fold change (LC) (Supplementary Table 3). GSEA revealed the upregulation of genes involved in morphogenesis and development of different tissues, as well as extracellular matrix components and signaling pathways WNT and BMP (Fig. 3a). The downregulated categories consisted of presynaptic components, including the synaptic vesicle genes (Fig. 3b). In addition, we were specifically interested in the enrichment of genes involved in protein–protein interactions (PPI) with CASK (Pathway Commons), genes linked to ASD (SFARI scores 1, 2, and syndromic), and a compiled list of NDD genes (Supplementary Table 4). We detected significant enrichment of CASK PPIs in downregulated genes (*p* = 0.003, FDR = 0.003, GSEA, Fig. 3c, Supplementary Table 5) and no enrichment in upregulated genes (*p* = 0.17, FDR = 0.25, GSEA, Fig. 3c, Supplementary Table 5). Core enrichment genes are within a densely connected CASK PPI network, including MINT1 (encoded by *APBA1*), which is one of the tripartite binding partners of CASK (Fig. 3d). We separately looked at the nuclear interaction partners TBR1, CINAP (*TSPYL2*), and BCL11A that were not included in the obtained CASK PPI dataset. We found downregulation of *TSPYL2* (LC = −0.37, p.adj. = 2E−3, DESeq2) and *BCL11A* (LC = −0.53, p.adj. = 1E−4, DESeq2), and the absence of *TBR1* expression in our model. Moreover, we detected a nominally significant enrichment of NDD genes in upregulated genes (*p* = 0.002, FDR = 0.25, GSEA, Fig. 3c, Supplementary Table 5). In addition, we performed GSEA enrichment for individual cases versus sex-matched controls (Fig. 3c, Supplementary Tables 5–7), and found that CASK PPI and NDD genes were more enriched in the pooled analysis, indicating that this approach allowed us to detect consistent expression changes, independent of genetic backgrounds. Enriched upregulation is detected from sex chromosome bands Xp22 and Xq22, as well as cytoband 1q44 in MICPCH_CASK_dup4/5_ (FDR of 0.003, 0.005, and 0.002, respectively), and downregulated genes originating from cytoband Yq11 were enriched in ASD_CASK_SS_ (FDR = 0.021).

In a complementary approach to the case-vs.-control analysis, we overlapped the most significant DEGs of case vs. sex-matched control analyses (Fig. 3e). Overrepresentation analysis in GO-term biological processes confirmed extracellular structure organization (*p* < 0.0001, FDR < 0.0001, WebGestaltR, Supplementary Table 8) and synaptic vesicle cycle genes (*p* < 0.0001, FDR = 0.0002, WebGestaltR, Supplementary Table 8) as the most significant upregulated and downregulated process, respectively. The results from pathway analysis demonstrated that *CASK*-mutant maturing neurons are deficient in expression of presynaptic CASK-interacting proteins.

We performed siRNA-mediated knockdown of *CASK* to verify *CASK*-dependent expression changes during differentiation. We transfected male control cells after 1 day of differentiation with a pool of CASK-specific siRNAs or nontargeting control siRNA. Knockdown was efficient with a significant reduction of CASK mRNA and protein at day 28 (Supplementary Fig. 4i, j). GSEA of DEGs confirmed changes in synaptic vesicle pathway and extracellular matrix compounds (Supplementary Fig. 4k). Similar to DEGs from *CASK-*mutation carrier cell lines, NDD genes were nominally enriched in upregulated genes after *CASK* KD (*p* = 0.031, FDR = 0.58, GSEA, Supplementary Table 9). In contrast to reduced expression of synaptic vesicle genes in the *CASK*-mutation carrier neurons, the genes underlying synaptic vesicle enrichment in response to CASK KD were upregulated. The conflicting direction of expression changes may be due to priming effects of reduced CASK in NES cells before differentiation.

To test if the dysregulation of presynaptic genes results in aberrant presynaptic development, we assessed synapse morphology in maturing neuronal cultures through immunofluorescence co-staining of pre- and postsynaptic markers Synapsin-1/2 and Homer-1, respectively (Fig. 3f). Synapsin-1/2 is part of the CASK PPI network (Fig. 3d), and the expression of SYN1 gene was downregulated (LC = −1.08, adj.p = 3.03E−13, DESeq2). The Homer-1 gene, *HOMER1* also showed downregulation (LC = −0.33, adj.p = 0.002, DESeq2). The number of Synapsin-1/2-positive presynaptic and Homer-1-positive postsynaptic regions did not reveal any difference between cell lines (Supplementary Fig. 5a). Similarly, the number of colocalizing regions, indicative of functional synapses, did not differ. While the Homer-1 particle size was comparable between cell lines (Fig. 3g), the mean Synapsin-1/2 particle size was smaller in ASD_CASK_SS_ compared to male controls and MICPCH_CASK_dup4/5_ (*p* = 0.021 and *p* = 0.014, respectively, ANOVA post hoc Tukey, Fig. 3h). Although dysregulation of presynaptic pathways was detected in the MICPCH_CASK_dup4/5_ cell line, the presynaptic Synapsin-1/2 size was comparable to controls. The size differences indicated that reduced *SYN1* expression affected presynaptic Synapsin-1/2 size in ASD_CASK_SS_. Differences in Synapsin-1 particle size and a more severe cellular phenotype in ASD_CASK_SS_ are in line with the results obtained for CASK puncta.

### E/I balance is affected by reduced CASK levels

**Fig. 4:**
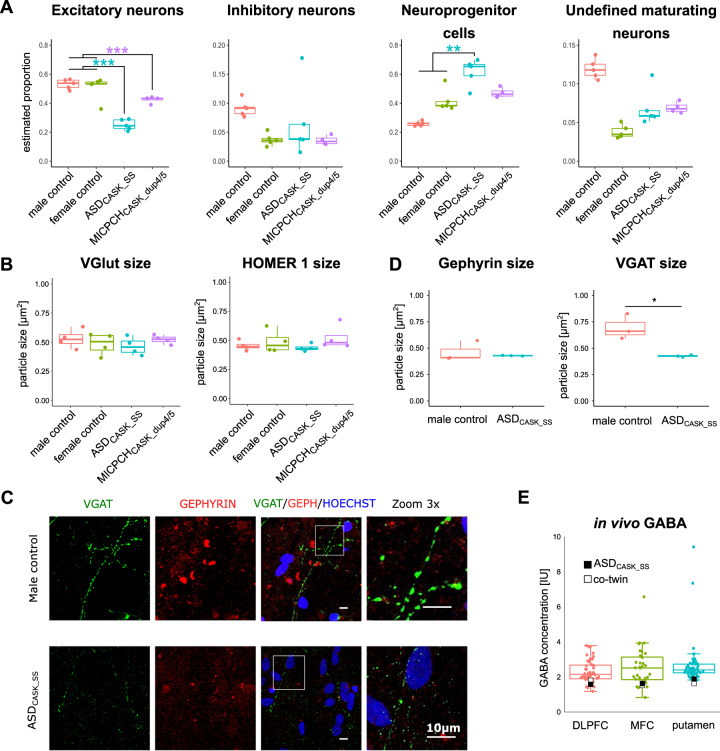
Presynaptic effect is limited to inhibitory VGAT presynaptic marker. **a** Deconvolution of cell-type populations underlying bulk RNA-sequence samples from all cell lines. **b** Quantification of VGlut and Homer-1 particle size (*n* = 4 per cell line). **c** Representative confocal microscopy images of neurons differentiated for 28 days and immunostained for inhibitory VGAT (green), Homer-1 (red), and Hoechst (blue). Scale = 10 µm. **d** Quantification of VGAT and Homer-1 particle size (*n* = 3 per cell line). Statistical differences between cell lines were calculated using ANOVA with post hoc Tukey HSD. *p < 0.05, **p < 0.01, ***p < 0.001. Asterisks are color-coded according to case cell lines. **e** In vivo concentration of GABA in the DLPFC, MFC, and putamen of ASD_CASK_SS_ and his cotwin in relation to a typical developed control group.

We explored the effect of the *CASK* mutations on the identified cell types using the BSEQ-sc analysis pipeline for gene expression deconvolution^40^. We used the cell-type markers generated from the single cell RNA sequencing data from MICPCH_CASK_dup4/5_ cells to predict cell-type proportions underlying the bulk RNA transcriptomes. In our analysis, the estimated frequency of inhibitory GABA and undefined neuronal subtypes was unaffected in mutation carrier cell lines (Fig. 4a). Excitatory neurons were predicted with a lower frequency in both case cell lines, and NPCs were predicted with a higher frequency in ASD_CASK_SS_. In accordance with changes in protein expression and morphological features, ASD_CASK_SS_ showed a stronger effect than MICPCH_CASK_dup4/5_. The deconvolution of the *CASK* KD transcriptome predicted no change in cell-type proportions (Supplementary Fig. 5b).

Differences in cell-type proportions should be detectable on a morphological level. We performed immunofluorescence co-staining of the excitatory postsynapse marker Homer-1 with the presynaptic markers VGlut and VGAT as a proxy for excitatory and inhibitory presynapses, respectively. We had detected downregulation of *SLC17A6* encoding for VGlut (LC = −1.38, adj.p = 2.44E−8, DESeq2) and *SLC32A1* encoding for VGAT (LC = −2.44, adj.p = 1.09E−37, DESeq2) in the bulk RNA sequencing comparisons. VGlut particle number and size were comparable between cell lines (Fig. 4b, Supplementary Fig. 5c). The consistent number of VGlut particles per nuclei indicated comparable numbers of excitatory synapses. Staining of the inhibitory marker VGAT showed significant decrease in particle size between ASD_CASK_SS_ and controls (ASD_CASK_SS_ vs. female control *p* = 0.005 vs. male control *p* = 0.009, ANOVA post hoc Tukey, Supplementary Fig. 6a, b). Co-staining of the inhibitory postsynaptic marker Gephyrin with VGAT in ASD_CASK_SS_ confirmed reduced VGAT particle size (ASD_CASK_SS_ vs. male control *p* = 0.018, ANOVA post hoc Tukey, Fig. 4c, d). The size of colocalizing VGAT and Gephyrin particles, indicative of functional inhibitory synapses, was not significant (ASD_CASK_SS_ vs. male control *p* = 0.067, ANOVA post hoc Tukey, Supplementary Fig. 5d). In agreement with previous findings for CASK and Synapsin-1/2 particles, the number of presynaptic particles was unaffected in case cell lines (Supplementary Fig. 5c, d). Reduced VGAT particle size could indicate reduced inhibitory synapse size. Comparable VGAT presynaptic particles per nuclei indicate that inhibitory synapse numbers are comparable.

To compare our iPSC-derived neuronal data of reduced VGAT staining in inhibitory synapses to in vivo neurotransmitter concentrations, we investigated proton magnetic resonance spectroscopy ([1H]MRS) data, available for twin pairs including ASD_CASK_SS_^43^. We analyzed the GABA concentration in individuals without NDDs and obtained data for the dorsal medial prefrontal cortex (DLPFC, control *n* = 44), medial frontal cortex (MFC, control *n* = 33), and putamen (control *n* = 45) (Supplementary Fig. 6a–d). The GABA concentration of the ASD_CASK_SS_ individual ranked in the lowest percentile in the DLPFC (*z* = −1.16) and putamen (*z* = −0.67) and in the 9th percentile of the MFC (*z* = −0.86). GABA in the mutation carrier cotwin ranked similarly in the three brain regions (DLPFC: *z* = −0.81, putamen: *z* = −0.67, and MFC: *z* = −0.97, Fig. 4e).

**Fig. 5:**
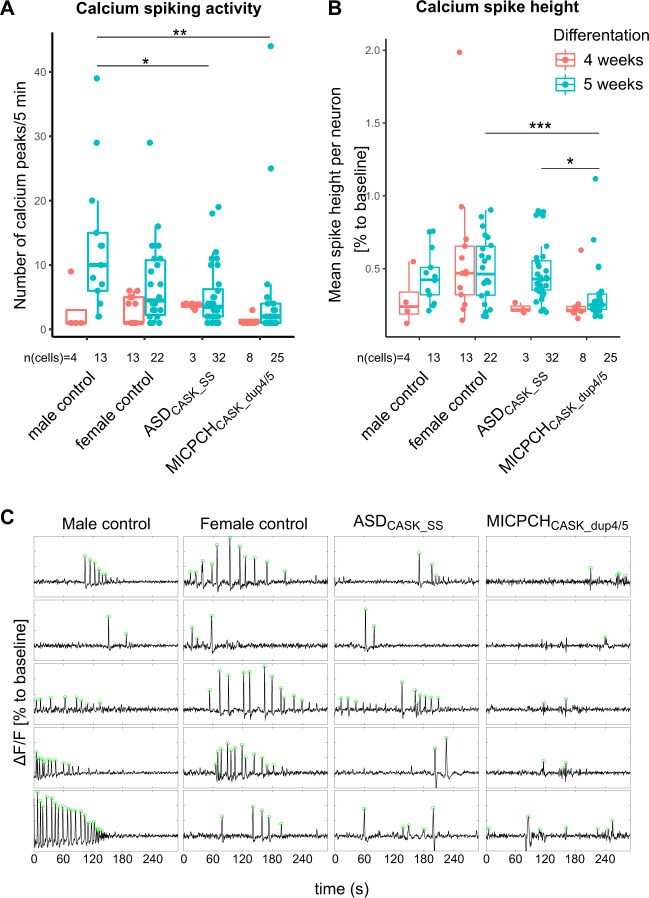
Neuronal activity measured by time-lapse calcium imaging. **a** Number and **b** height of calcium spikes in active neurons of mutation carriers and controls differentiated for 4 and 5 weeks. Number of total neurons below the *x*-axis. Biological replicates at 4 weeks: female control (*n* = 2), male control (*n* = 2), ASD_CASK_SS_ (*n* = 1), and MICPCH_CASK_dup4/5_ (*n* = 2). Five weeks: female control (*n* = 2), male control (n = 2), ASD_CASK_SS_ (*n* = 5), and MICPCH_CASK_dup4/5_ (*n* = 2). Statistical differences between cell lines were calculated using pairwise Wilcoxon Rank Sum test followed by Bonferroni correction. *p < 0.05, **p < 0.01. **c** Representative 5-min traces of active neurons after 5-week differentiation with called peak locations (green).

Finally, we investigated spontaneous neuronal activity in the neuronal cultures. We imaged intracellular calcium release in 4- and 5-week-old neuronal cultures and compared calcium-signaling events between the cell lines. We identified spontaneously active neurons in 4-week-old cultures and observed an increased rate of calcium signals after 5 weeks (Fig. 5a, c, Supplementary Fig. 7g). No differences were detected between cell lines after 4 weeks of differentiation. At 5 weeks, ASD_CASK_SS_ and MICPCH_CASK_dup4/5_ showed less activity as compared with the male control (ASD_CASK_SS_ *p* = 0.0266, MICPCH *p* = 0.0019, Bonferroni-corrected Pairwise Wilcoxon Rank Sum Test). In contrast to the mild cellular phenotype observed for MICPCH_CASK_dup4/5_, we observed aberrant calcium spike height (vs. female control *p* = 0.035 vs. ASD_CASK_SS_ *p* < 0.001, Bonferroni-corrected Pairwise Wilcoxon Rank Sum Test, Fig. 5b). The rise and decay times are unaffected in ASD_CASK_SS_ and MICPCH_CASK_dup4/5_ (Supplementary Fig. 7e, f). In agreement with transcriptomic and morphological results, neuronal network activity was affected in ASD_CASK_SS_ with decreased firing rate and unaltered spike characteristics.

## Discussion

*CASK*-related disorders have emerged as an important genetic diagnosis for children with NDDs ranging from severely affected MICPCH patients to individuals with ASD^1,4,6,7^. The role of CASK as an essential player in synapse formation was reported previously using model organisms^13,16,17^; however, there is limited information of how the different *CASK* genetic variants found in humans affect the neuronal development. Therefore, we studied the consequences of two different mutations affecting *CASK* from individuals diagnosed with MICPCH and ASD. We utilized an iPSC- derived model to investigate the mutation effects during early neuronal maturation with detailed molecular, cellular, and functional characterization. We show that even slight changes in CASK expression in humans lead to dysregulation of the formation of presynapses, especially in inhibitory neurons. We suggest that the revealed presynaptic phenotype could be underlying the social difficulties seen in individuals affected by *CASK*-related disorders, and that there are other mechanisms leading to the more profound brain malformations seen in more severely affected patients.

Using transcriptomic profiling of maturing neurons from *CASK-*mutation carriers and controls, we demonstrated that reduced CASK levels affected the transcription of presynaptic genes with significant enrichment for CASK-interacting partners. Dysregulation of CASK-interacting proteins in synapse gene networks is in line with transcriptomic profiling in heterozygous *Cask*-knockout mice^51^. Importantly, downregulation of, e.g., *NRXN* family of genes, *STXBP1*, and *SYT1* in *CASK-*mutation carriers, provides a phenotypic link to other NDD genes. For instance, *NRXN1* is one of the most studied NDD genes in which homozygous mutations cause Pitt–Hopkins-Like syndrome 2 characterized by NDDs, such as ID and ASD^52^, and heterozygous deletions predispose to several NDDs, psychiatric disorders, as well as congenital malformations^53,54^. Mutations in *STXBP1* were first described in patients with early infantile epileptic encephalopathy, and are a common cause of epilepsy and encephalopathy with ID^55,56^. *SYT1* mutations in Baker–Gordon syndrome cause infantile hypotonia, ophthalamic abnormalities, hyperkinetic movement disorders, motor stereotypies, and DD^57^. Disruption of this protein network in the developing presynapse could be underlying the behavioral phenotypes underlying *CASK*-related NDDs. In addition to the presynapse, CASK has been implicated in functions within the dendrites, postsynapse, and neuronal nucleus. Although we observed the strongest enrichment in the presynapse, functional interaction partners of CASK in the other neuronal compartments were also dysregulated. We observed upregulation of *CNTNAP2* and *SDC2*, which form complexes with CASK in dendrites to regulate postsynaptic development^50,58^. *CNTNAP2* is linked to Pitt–Hopins-Like syndrome 1 and increased ASD risk^52,59,60^. In addition, we observed downregulation of nuclear interacting partners *BCL11A* and *TSPYL2*, which have been linked to ID and ASD, respectively^22,23^. Taken together, *CASK* mutations cause dysregulation of NDD-related networks with a strong effect on presynapse development and putative impact on additional aspects of neuronal functions. Interestingly, we also illustrate upregulation of many developmental pathways in our analyses, such as mesenchyme, face and head development, as well as WNT signaling. Future studies should investigate these pathways in more detail and characterize how they might be linked to *CASK*-related phenotypes, including microcephaly, scoliosis, and abnormalities of the eye and skin development^1^.

One benefit of the neuronal differentiation scheme used here was that we could investigate the effects on both inhibitory and excitatory neurons, and demonstrate that the transcriptional changes translate to reduced presynaptic size of inhibitory neurons as measured by Synapsin-1/2 and the inhibitory marker VGAT. The reduced presynaptic size, as measured by the selected markers and CASK puncta, may indicate aberrant synaptogenesis and synaptic function. In turn, this could lead to E/I imbalance. Earlier studies using electrophysiological measures in transgenic mice demonstrated decreased frequencies of mIPSCs and increased mEPSCs in adult CASK-deficient neurons^16,17^, establishing aberrant E/I balance as a phenotype underlying CASK deficiency. In addition, Mori et al. (2019) detected a decreased expression of the glutamate receptor GluN2B (encoded by *Grin2b*) in CASK-deficient neurons and concluded that postsynaptic CASK deficiency has an influence on E/I balance. In line with the previous findings, we detected reduced *GRIN2B* expression in ASD_CASK_SS_ during neuronal maturation. However, we detected an unaffected number and size of excitatory VGlut synapses in contrast to the reduction in inhibitory presynaptic size, which together suggest that reduced inhibitory signaling is the primary mechanism underlying E/I imbalance in CASK-deficient neurons. Moreover, the low GABA concentration in all assessed brain regions of ASD_CASK_SS_ and his cotwin could indicate that the inhibitory synapse effect is persistent during postnatal development. On the in vitro level, we observed reduced neuronal activity in the mutation carrier neurons. Changes in neuronal proportions, as suggested by the proportion estimates, could explain changes in neuronal network activity. However, consistent numbers of VGlut and VGAT punctae suggested stable cell-type proportions across cell lines. The observed decrease in VGAT size at GABAergic synapses could explain reduced bursting frequency. While a reduced inhibitory frequency would be expected to increase network activity, reduced bursting complexity could explain the reduced number of neuronal calcium events. In addition, GABA neurotransmitter function changes from excitatory to inhibitory during neural development and may have had an excitatory function in our model system^61^. Electrophysiological characterization of inhibitory and excitatory postsynaptic currents (IPSC and EPSC) in ASD_CASK_SS_ and MICPCH_CASK_dup4/5_ neurons during neuronal maturation is necessary to link the observed changes in presynaptic marker size to changes in synaptic strength. E/I imbalance caused by aberrant neuronal firing was described for a variety of genetic mutations underlying NDD phenotypes. Studies investigating *NRXN1* showed that similar to *Cask* KO mice, *Nrxn1* KO mouse neurons remain with normal postsynaptic current amplitudes, but in contrast to *Cask* KO showed decreased mEPSC frequencies^62^. In human embryonic stem cell-derived neurons with heterozygous *NRXN1* LoF mutation, increased CASK protein levels were observed together with decreased EPSC frequencies^18^ and homozygous deletions impair neuron maturation^63^, indicating that CASK-related pathways affect E/I balance. In a recent study of isogenic human excitatory neurons disrupted for ASD-relevant genes, electrophysiological assessment demonstrated reduced spontaneous EPSCs and neuronal bursting in the presence of homo- or hemizygous mutations of *ATRX*, *AFF2*, *KCNC2*, *SCN2A*, and *ASTN2*^64^. Another study detected reduced neuronal firing in iPSC-derived neurons of eight individuals with idiopathic ASD^65^ and detected a correlation of reduced network complexity to behavioral and cognitive phenotype of the donors^66^. Moreover, reduced GABA concentration, as seen in the ASD_CASK_SS_ brain, was reported in groups of children with ASD^67^. Taken together, these findings show that *CASK*-related NDDs share the E/I imbalance as pathological mechanisms with other ASD- and NDD-risk genes.

As an in vitro stem cell-based study, our results provide limited information in respect to the precise neurodevelopmental period and brain regions in which the observed effects contribute to the patient phenotype. We analyzed a short window of neuronal maturation and observed aberrant inhibitory presynaptic development in differentiated neurons. Longitudinal studies would be required to determine if these effects are persistent or represent delayed maturation. Postsynaptic effects, as well as effects at the excitatory neurons, have been described in models of *CASK* deficiency and certainly contribute to the phenotype range and severity in cases of *CASK*-related disorders. Our study is limited to the characterization of the effects of reduced CASK protein levels, and it remains elusive how cellular phenotypes of *CASK* missense mutations compare. Thus, our results cover a limited part of the underlying molecular etiology.

In addition to the effects seen overall for the neuronal cultures, our study shows intriguing findings from the two unique mutations affecting *CASK*. The hemizygous splice-site mutation detected in an autistic individual and his mild symptomatic monozygotic cotwin reduced expression of CASK_WT_ and caused splicing of two mutant mRNA isoforms. We excluded gain-of-function effects from mutant mRNA isoforms, as we detected only wild-type protein with similar cellular localization as in matched control neurons. Interestingly, we observed the rescue of the *CASK*_WT_ splicing by an unknown mechanism. RNA sequencing of ASD_CASK_SS_ neurons indicated that the mutated T/U is modified to the reference G nucleotide, suggesting that RNA editing is involved in the rescue mechanism. While reports on U-to-G RNA editing are rare, these mechanisms are shown to be relevant for synaptic transcripts and may be involved in the rescue of *CASK*_WT_ in the ASD_CASK_SS_^68,69^. As both monozygotic twins carry the splice-site mutation and exhibit differences in symptom severity, it is interesting to speculate that environmental stressors could influence the rescue mechanisms and thus lead to variable severity. We have earlier studied pre- and perinatal environmental exposures in the RATSS twin sample, including the ASD_CASK_SS_ case and his cotwin, and showed that uptake of essential metals zinc and manganese, and the neurotoxic metal lead, was associated with variable manifestation of autistic traits and ASD diagnoses^70^. These environmental stressors could potentially affect the RNA processing efficiency and mediate gene-environment interactions, leading to differences in outcomes of the twin pair^68,71,72^.

The instability of XCI has been reported in iPSCs and has hindered the cellular investigation of dominant X-linked genes in NDDs. In the MICPCH_CASK_dup4/5_ cells of the female donor, we observed mutant and wild-type *CASK* mRNA expression that likely resulted from XCI escape of the wild-type allele. Reports of XCI in adult human tissues did not indicate *CASK* as likely XCI escape gene^73^. Heterozygous *Cask*-knockout mice that recapitulate the MICPCH phenotype showed *Cask*_WT_ expression in 50% of neurons, suggesting that XCI is stable in *CASK*-negative neurons^17,74^. The stability of XCI during iPSC reprogramming varies between protocols, and activation of both X chromosomes with subsequent random silencing of one X has been observed^75^. The milder phenotype in females carrying *CASK* mutations with skewed XCI suggests that the proportion of *CASK*-deficient cells is crucial for phenotype severity^5,8^. The escape of *CASK*_WT_ in the MICPCH_CASK_dup4/5_ cells likely rescues the cellular phenotype in our in vitro model, and we were unable to capture all pathoetiological mechanisms in the case with MICPCH. Indeed, we consistently observed less severe cellular phenotypes in the MICPCH_CASK_dup4/5_ cells than ASD_CASK_SS_ cells, which is in strong contrast to the clinical phenotype severities of both disorders. This demonstrates the necessity to control for XCI pattern in iPSC studies of X-linked genes.

Currently, there are no drugs to treat *CASK*-related disorders. A recent clinical study assessing neurorehabilitation effects on three girls with MICPCH and *CASK* mutations at the age of 2–4 years showed that the girls improved in motor skills, social interaction, and communication functions, indicating potential benefits of targeting the social aspects in *CASK-*related disorders^76^. As we demonstrate a specific effect on the inhibitory presynapse and reduced GABA concentration in the cortical and subcortical brain regions of ASD_CASK_SS_, it is intriguing to speculate on the effect of GABA modulators in *CASK*-related disorders. Especially GABA agonists could be tested, which have shown positive effects on social deficits in ASD, and are in clinical trials for Fragile-X syndrome^77–79^. Further development of drugs that target the presynapse and synaptic vesicle cycle could also be beneficial, but are currently not well explored^80^.

In conclusion, we show that reduced CASK protein levels affect presynaptic development and decrease inhibitory presynapse size, which may have consequences to E/I balance in developing neural circuitries. Aberrant E/I balance, and synaptogenesis are two common biological pathways underlying NDDs of different genetic origin. Future pharmacological and clinical studies on targeting presynapses and E/I imbalance could lead to specific treatments for *CASK*-related disorders.

## Supplementary information

Supplementary Information

Supplementary Table 3

Supplementary Table 4

Supplementary Table 5

Supplementary Table 6

Supplementary Table 7

Supplementary Table 8

Supplementary Table 9
