# Supplementary material for: Presynaptic dysfunction in CASK-related neurodevelopmental disorders
Source: Transl Psychiatry. 2020 Sep 14;10:312. doi: 10.1038/s41398-020-00994-0 (PMC7490425; doi:10.1038/s41398-020-00994-0)
Supplement: Supplementary file 1 — Supplementary Information [file 41398_2020_994_MOESM1_ESM.pdf]

## Supplemental information

## Supplementary Materials and Methods

### *Identification of mutation carriers*

The mutation carriers were recruited to the study from two different sources: The Roots of Autism and ADHD study in Sweden (RATSS) and Clinical Genetics at the Karolinska University Hospital. The comprehensive phenotyping procedures for the twin pair has been described elsewhere [1, 2] and included T1-weighted spoiled gradient echo anatomical MRI scans (176 slices, TR=8.2s, FOV=240; acquired with a 3 Tesla MR750 GE scanner). Phenotyping, genotyping and clinical assessment of the girl with MICPCH was described earlier [3]. Written informed consent was obtained from affected individuals and their parents prior to the study. The study was approved by the regional and national ethical boards in Sweden, and it has been conducted in accordance with the Declaration of Helsinki for medical research involving human subjects, including research on identifiable human material and data.

### *Whole-exome sequencing and data processing*

For the identification of the genetic alterations, we performed both microarray analysis (for both cases) and whole exome sequencing (for the twin pair and their parents). For *de novo* variant detection, the parents were included in the genetic analyses. DNA was extracted from whole blood and saliva samples using standard methods. The detection of CNVs has been described earlier [3, 4]. For WES, 3 µg DNA (200 ng for the mother) was used for library preparation with Agilent SureSelect Human All Exon V5 kit followed with quantification of the libraries using KAPA Biosystems' Library Quantification according to the manufacturer's recommendations. The sequencing was performed on Illumina HiSeq 2500 using the Rapid SBS Kit v2 chemistry. After sequencing, base calling was done with Illumina Casava pipeline v1.8.2, and reads were mapped to the hg19 reference sequence using the Burrows-Wheeler Aligner (BWA) v0.5.9. Duplicate reads were marked and removed using Picard v1.79 tools. Variant calling, local realignment around indels and base quality score recalibration were conducted with Genome Analysis ToolKit (GATK) v1.1-28. Basic variant calling was followed by annotation using a custom pipeline at the Center of

Applied Genomics (TCAG) based on Annovar that creates a merged file for the twin pair and parents [5]. Population frequency for each variant was based on the 1000 Genomes Project, the Exome Variant Server hosted by NHLBI (Exome variant server), 69 Complete Genomics public genomes and The Exome Aggregation Consortium (ExAC). Additional information about putative pathogenicity and disease association of variants were based on different databases such as dbSNP, Clinvar, Online Mendelian Inheritance of Man (OMIM), and Clinical Genomics Database (CGD). Reported mutation sites were transferred to hg38 using LiftOver (UCSC Genome Browser).

*Identification of putative risk variants underlying the NDD phenotype*

For the analysis of putative NDD risk variants seen in both $ASD_{CASK\_SS}$ and his co-twin, only rare variants (< 1.0% in the population) were selected. The remaining variants were filtered based on the impact of the variant and association of the gene to NDDs. In brief, the genes affected by the prioritized variants were compared against a list of known ASD and ID genes [6] and genes implicated in neurodevelopmental/behavioral phenotypes in human or mouse from the human phenotype ontology (HPO) [7]. Additionally, a list of genes implicated in ASD from two large scale sequencing studies [8, 9] was compiled and used in the analysis. The mode of inheritance for the genes was curated from the OMIM and CGD databases. Further prioritization was made based on the predicted pathogenicity of the variants; *de novo* variant, LoF (loss of function, i.e. stop-gain, frameshift, essential splice site alterations, or missense near splice-site) and missense variants were selected for further evaluation. Final categorization was made based on the criteria from the American College of Medical Genetics and Genomics (ACMG) [10].

To validate genomic variants, genomic regions were PCR amplified using sequence specific primers with HotStarTaq Polymerase Kit (Qiagen # 203203). PCR primer sequences used to amplify the CASK_SS variant: AGAAAATCCCTTGTCTGATGA (Fw) and CCTGCCATAAAAATCCACTC (Rv). DIAPH1 variant: TTACTTACCCCCAACACAAAC (Fw) and TTACTGAGATGGAGAGCTTGC (Rv). The PCR products were purified, and Sanger sequenced.

*Generation of neuroepithelial stem cells*

Human iPSC cells were derived for the *CASK* mutation carriers diagnosed with MICPCH and ASD, using a previously described procedure [11]. In summary, fibroblasts were obtained from skin biopsies and reprogrammed to iPSC using Sedai virus transfection of the Yamanaka factors *OCT4, SOX2, KLF4,* and *cMYC*. Human iPS cells were maintained in xenofree and defined conditions using, substrate Laminin521 (BioLamina #LN521), and Essential E8 media (ThermoFisher Scientific # A1517001). Quality of iPSC induction was done with NANOG and OCT4 staining (Figure S6A) and gene-expression based pluripotency test [12]. Genetic identity was controlled with karyotyping (Figure S6B) and short-tandem repeat analysis of 20 markers.

iPS cells were passaged as single cells by TrypLE Select (1X) (Gibco # 12563029) once they reached 80-90% confluence. Dual-SMAD inhibition was applied to derive NES cells from human iPS cells as described in Chambers SM et al. 2009 [13]. On the first day of neural induction, iPS cells were seeded at a density of 40 000 cells/cm$^2$ on LN521 substrate and in Essential E8 medium. Day 1-5 of Neural Induction, E8 medium was replaced with KRS-medium including the human recombinant Noggin (hNoggin, PeproTech #120-10C) at a final concentration of 500ng/ml, SB431542 (StemCell Technologies #72232) at a final concentration of 10 μM and CHIR99021 (StemCell Technologies #72052) at a final concentration of 3,3 μM. The media was replaced daily. Day 6-12, KOSR media was gradually replaced increasing amounts of N2-media (25%, 50%, 75%), according to Chambers at al 2009, maintaining the factors hNoggin at a final concentration of 500 ng/ml and CHIR99021 at a final concentration of 3,3 μM. At day 12 of neural induction, cells were dissociated by TrypLE Express, and seeded in DMEM/F12+Glutamax medium (Gibco) supplemented with 0.025X B-27 (Gibco), 1X N-2 (Gibco), 10 ng/ml recombinant human FGF (Gibco), 10 ng/ml recombinant human EGF (Peprotech) and 10 U/ml Penicillin/Streptomycin (Gibco). Cells were seeded at a density of 250 000-280 000 cells/cm$^2$, on plastic surface double-coated with 20 ug/ml poly-ornithine (Sigma Aldrich) and 1 ug/ml laminin (Sigma Aldrich). The cells were further passaged at a density of 40 000 cells/cm$^2$. Half of the medium was changed daily.

*Differentiation of NES cells*

NES cells were seeded on plastic surface, pre-coated with 20 ug/ml poly-ornithine (Sigma Aldrich) and 1 ug/ml laminin (Sigma Aldrich) in DMEM/F12+glutamax medium (Gibco) supplemented with 0.5X B-27 (Gibco), 1X N-2 (Gibco) and 10 U/ml Penicillin/Streptomycin (Gibco). For

immunofluorescence, neurons were seeded on a glass surface, pre-coated with 200 ug/ml poly-ornithine (Sigma-Aldrich), and 4 ug/ml laminin (Sigma-Aldrich). Cells were maintained in a 5% $CO_2$ atmosphere at 37°C. Two third of the medium was changed every second day of differentiation with 0.4 ug/ml laminin added. Neurons were differentiated for 8, 16 or 28 days. Cells harvested for time point day 0 were cultured for two days in NES cell medium.

*RNA extraction and RT-qPCR*

Cells were lysed in TRIzol reagent (Invitrogen), and RNA was isolated using the ReliaPrep RNA Cell Miniprep (Promega #A1222). The RNA was reverse transcribed using iScript cDNA Synthesis Kit (BioRad) and cDNA quantified with SsoAdvanced Universal SYBR Green Supermix (BioRad) following manufacturer protocols on a CFX96 thermal cycler (BioRad). CFX Manager software was used to record amplification curves and to determine Ct values. RT-qPCR reactions were performed in technical triplicates. We calculated the ΔCt to the GAPDH housekeeping gene and $^{\Delta\Delta}$Ct to control cell lines. We used three biological replicates obtained in individual experiments, if not stated otherwise in the figure legends. Statistical significance between cell lines was determined with ANOVA and posthoc Tukey HSD in R.

*Immunodetection using a capillary western blot*

Cells were dissociated in extraction buffer (50 mM Tris-HCl, 100 mM NaCl, 5 mM EDTA, and 1 mM EGTA) using plastic cell scraper and lysed with 6 short sonication bursts at 36% amplitude (Vibra-Cell VCX-600, Sonics). Total protein yield was quantified with Pierce BCA assay (Thermo Fisher), and 250 µg/ml protein was loaded for Simple-Western WES (ProteinSimple) quantification. Antibodies were used to detect the proteins CASK (1:500 Novus Biologicals NBP2-41181), beta-actin (1:100 Abcam ab8227), and GAPDH (1:5000 Sigma G9545). The Compass Software For Simple Western (Version 4.0.0) was used to identified peaks at known molecular weights. In the chromatogram, the peak area was used for protein quantification. We normalized CASK protein for housekeeping protein. For time point days 0, 8, and 16, we obtained two biological replicates and three biological replicates for time point 28 and siRNA-mediated

knockdown of CASK. Statistical significance between cell lines was determined with ANOVA and posthoc Tukey HSD in R.

*Immunofluorescence*

Cells were cultured on glass coverslips for the indicated differentiation time. They were fixed for 20 min in 4% formaldehyde, rinsed with 1X TBS and blocked with blocking buffer (1X TBS, 5% Donkey Serum, 0.1% Triton X-100) for 1h. The slides were incubated with the primary antibodies diluted in blocking buffer overnight at 4°C. Primary antibodies used are CASK- NBP2-41181, 1:500 (Novus bio), MAP2-M2320, 1:500 (Sigma Aldrich), VGLUT1-135304, 1:250 (Synaptic systems), Homer-1-160011, 1:250 (Synaptic systems), VGAT-131003, 1:500 (Synaptic systems), Synapsin-1/2-106006, 1:500 (Synaptic systems), Gephyrin-147021, 1:250 (Synaptic systems) Nestin-MAB5326-KC, 1:1000 (Merk-Millipore), SOX2-AB5603, 1:1000 (Merk-Millipore). Slides were thoroughly washed with 1X TBS and incubated for 2 hours with secondary antibodies, and 1:500 Hoechst diluted in blocking buffer. The coverslips were washed and mounted with Diamond Antifade Mountant (Thermo Scientific). All images were taken with LSM 700 Zeiss Confocal Microscope, with 63x magnification at 1024 x 1024 pixel [pxl] resolution, resulting in an aspect ratio of 0.099233 µm per pixel.

CASK puncta, as well as nuclei, were counted with the ImageJ Particle Analyzer, after converting fluorescent images to binary images (threshold function “Moments” for CASK and “Default” for nuclei). Quantification of puncta number and size was done in R. Synaptic marker particle size and number were quantified with the ImageJ plugin Synapse Counter, using default settings [14].

*siRNA-mediated gene knockout*

NES cells were seeded for differentiation and the next day transfected with 0.5µM Accell SMARTpool siRNA targeting *CASK* mRNA (Dharmacon #E-005311-00-0010) or 0.5µM Accell Green Non-targeting siRNA (Dharmacon #D-001950-01-20) according to standard protocol. Three biological replicates were harvested for each protein and RNA analysis.

*Single-cell RNA sequencing*

Cells were dissociated from culture surface with 2 min trypsin incubation and subsequent trypsin inhibition. After 3 minutes' centrifugation at 300 rcf the cells were resuspended in cold Dulbecco's PBS and single cells sorted by size into lysis-buffer using BD FACS Aria III. Smart-Seq2 library preparation and sequencing were done with the Eukaryotic Single Cell Genomics (ESCG) facility in the SciLifeLab, Stockholm [15]. 384 sorted wells were sequenced with a total of 209.6 M reads and an average sequence depth of 550,000 reads per cell. Sequence reads were demultiplexed using deindexer and aligned to reference genome GRCh38/hg38 (STAR). Approximately 80% of uniquely aligned reads aligned to the genome, 40% to exons and 30% to introns (Figure S3A-B). The individual counts per gene and cell were reported in a count matrix (GSE140572) and used for further analysis. The scImpute package was used to calculate dropout expression values from the count matrix [16]. Cells with less than 50,000 read counts in less than 2,000 genes were removed from analysis. Differential gene expression and cell clustering were done using the Seurat package [17]. To obtain mutant CASK reads, we created a custom genome with STAR that included the cDNA sequence of the CASK gene with the duplicated exons 4 and 5 and re-aligned all sequence reads. Exon 5 to exon 4 spanning sequence reads were analyzed using vcftools.

*Bulk RNA sequencing*

We extracted RNA samples of five biological replicates per cell line that were obtained in independent experiments. Samples were delivered to NGI Sweden for library preparation and sequencing. In short, strand-specific RNA libraries were prepared using Tru-Seq, including ribosomal depletion (Illumina). Samples were sequenced on NovaSeq6000 (NovaSeq Control Software 1.4.0/RTA v3.3.3) with a 2x151 setup using 'S1' flow cell mode. The Bcl to FastQ conversion was performed using bcl2fastq_v2.19.1.403 from the CASAVA software suite. The quality scale used is Sanger / phred33 / Illumina 1.8+. The NGI-RNASeq pipeline was used to trim reads (TrimGalore), align to reference genome GRCh38/hg38 (STAR), perform quality control (FastQC, RSeQC, dupRadar, MultiQC) and count gene reads (featureCounts). We obtained on average 50.4 million reads per sample with a minimum of 96.9% reads aligned to protein-coding

regions. Sample read counts are supplied in GSE140572. Replicates of each cell line cluster together with the exception of one MICPCH replicate that was removed from analysis. Read alignment at the CASK gene locus were visualized in the Integrative Genomics Viewer (IGV v2.5.3).

Differential gene expression was calculated from gene counts using DESeq2 (v1.24.0) in R. To determine a differential expression for individual cases, we used passage and cell lines as independent variables and compared $ASD_{CASK\_SS}$ with male control and $MICPCH_{CASK\_dup4/5}$ with female control. Top ranking genes for comparison between $ASD_{CASK\_SS}$ and $MICPCH_{CASK\_dup4/5}$ were filtered at an adjusted p-value of 1E-5 (Benjamin-Hochberg adjusted), with more than 20 reads and log2 fold change (LC) higher than 0.5. The Venn diagram was visualized in R using the VennDiagramm package (v1.6.20), and over-representation analysis was performed using WebGestaltR (v0.4.2).

*Curation of NDD gene list*

A list of known ASD and NDD genes were obtained from three online databases: Simons Foundation Autism Research Initiative (SFARI, https://gene.sfari.org/), DatabasE of genomiC varIation and Phenotype in Humans using Ensembl Resources (DECIPHER, https://decipher.sanger.ac.uk/ddd#overview), and Human Phenotype Ontology (HPO, https://hpo.jax.org/app/). All genes from DECIPHER, category 1, 2, and syndromic genes from SFARI and behavioral abnormality (HP:0000708) and cognitive impairment (HP:0100543) genes from HPO were included in the NDD gene list. Additionally, we included genes implicated in ASD, intellectual disability (ID) and developmental delay (DD) from five recent whole genome/exome sequencing and chromosome microarray studies [6, 18-21]. The date of data access is included in Table S4.

*Gene Set Enrichment Analysis*

The unfiltered gene expression list was ranked by fold-change expression changes. Gene set enrichment analysis was performed using the GSEA desktop software (Version 3.0) [22]. We used weighted enrichment statistics and included pathway gene set sizes between 3 and 250 genes.

Pathway gene sets were obtained from Reimand et al. [23]. Enriched categories were visualized in Cytoscape (v3.7.0) with Enrichment Map (v3.1.0) and annotated with AutoAnnotate [24, 25].

Specific GSEA was performed on CASK PPI (downloaded from PathwayCommons; PCViz: CASK on 08.Oct.2019), the NDD gene list, and SFARI sub-list. Specific GSEA was performed on ranked gene lists split into up- and downregulated genes. The CASK PPI network was visualized in Cytoscape.

### *Deconvolution*

The deconvolution of bulk RNASeq data was done using the BSEQsc package [26]. BSEQ-sc uses cell type specific marker genes from single-cell RNA transcriptomes to predict cell type proportions underlying bulk RNA transcriptomes. Deconvolution was done on increasing numbers of significant genes, and the predictions were stable when using 10 or 20 most significant marker genes. Statistical differences in the estimated cell proportions between patient and both control cell lines were done using ANOVA and posthoc Tukey HSD test.

### *Calcium Imaging*

Calcium events from neuronal activity last for 0.5 to 1 s and may be caused by individual action potentials or bursts of several action potentials [27, 28]. Intracellular calcium recordings were performed using a Fluo-8 AM dye (AAT Bioquest). A dye stock was prepared containing 0.3 mM Fluo-8 AM and 3.3% Pluronic F-127 (ThermoFisher Scientific) and mixed thoroughly. Stock was added to the cell culture media to a final dilution of 5 µM Fluo-8 and 0.05% Pluronic F-127. The medium was carefully exchanged, and cells were incubated for 45 min at 37°C with 5% $CO_2$.

Then culture dishes were transferred to an upright Zeiss Examiner.D1 microscope with a pE-300 light source (CoolLED) and a prime 95B camera (Photometrics). After an adjustment period of 5 minutes, time-lapse videos (20Hz, 40ms exposure, 5 min) were recorded in two random spots on the dish using a Zeiss 40x water immersion objective with an NA of 1. Recordings were performed using a windows computer running Micro-Manager (Open Imaging) [29].

Image time series were pre-processed to balance signal loss from fluorescence decay using the ΔF/F method in ImageJ [30]. Regions of interest (ROI) at active neurons with high variability of calcium signal were manually identified. Mean gray values of ROIs were recorded over the 5-minute pre-processed time series to obtain traces of neuronal activity. Traces were resampled to 2 Hz and smoothed using the python scipy.signal package. Peaks were detected using the PeakCaller Matlab software [31] with the following settings: 25% required rise, 25pts max lookback, 25% required fall, 25pts lookahead, Finite Difference Diffusion, 60 trend smoothness. Biological replicates at 4-weeks: Female control (n=2), male control (n=2), $ASD_{CASK\_SS}$ (n=1) and $MICPCH_{CASK\_dup4/5}$ (n=2). 5-weeks: Female control (n=2), male control (n=2), $ASD_{CASK\_SS}$ (n=5) and $MICPCH_{CASK\_dup4/5}$ (n=2). Active cells per cell line were pooled across replicates and statistical differences at 4 and 5 weeks were calculated using pairwise Wilcoxon Rank Sum test followed with Bonferroni correction.

*[1H]MRS*

Proton magnetic resonance spectroscopy ([1H]MRS) data was available from the EU-AIMS Longitudinal European Autism Project (LEAP) autism twin cohort, including $ASD_{CASK\_SS}$ [32]. MR data was acquired on MR750, 3 Tesla scanner (GE Healthcare, Milwaukie, USA), with 8 channel receiver array coil (in-VIVO inc.). To find small brain structures and to position the MRS voxel accurately and reproducibly a T1w 3D IR-SPGR (inversion-recovery spoiled gradient echo, resolution 1.0 x 1.0 x 1.2 mm) was acquired in the beginning of the protocol and the voxel position was confirmed thereafter by three-plane localizer images performed before every MRS scan. T1w MRI was setup according to ADNI2 protocol with flip angle 11°, te/tr = 3/ 7.3 ms, ti= 400 ms, number of slices 192 and bandwidth 244 Hz/px. MRS data was acquired with MEGA-PRESS pulse sequence, te/tr = 68/ 2000 ms, number of averages 192, phase cycle 8 steps and bandwidth 5 kHz for three voxels with volumes 19,3 mL (Medial PFC), 19.5 mL (Putamen-GPS), 13.6 mL (DLPFC) (Figure S6A). All voxels were positioned to maximize the grey matter (GM) content. CHESS water suppression and voxel OVS (6 outer volume suppression rf pulses) were applied. The gradient-echo shimming converged to the water linewidth of 10 ± 2 Hz for MPFC and Putamen-GPS and 6.9 ± 1 Hz for DLPFC.

GABA concentration of all three voxels was quantified from the MEGA-PRESS difference spectra using the "Gannet" GABA-MRS analysis toolkit, version 3.0 [33]. Fitting of the GABA peak is performed over a range of the spectrum between 2.79 and 3.55 ppm using a five-parameter Gaussian model and of the water peak using a Gaussian-Lorentzian function. Measuring GABA using MEGA-PRESS leads to co-editing of macro-molecules such as proteins that contribute to the edited GABA peak at 3.0 ppm. Metabolite values were scaled to water and expressed in institutional units (IU). Water-scaled GABA concentrations represent GABA as well as related macromolecules and are therefore referred to as GABA+ concentrations (Figure S6B). GABA concentrations were adjusted for the average proportion of partial GM and white matter (WM) volume in each voxel across all subjects. For tissue correction, the GABA concentration ratio α between GM and WM was set to 0.5 [34]. All spectra were visually evaluated for the quality of GABA fit. For the final analysis, 45 spectra from the Putamen (20 co-twins to individuals with NDDs), 33 from the MFC (11 co-twins to individuals with NDDs) and 44 from the DLPFC (19 co-twins to individuals with NDDs) of the control subjects could be included.

## References

1. Bölte S *et al.* The Roots of Autism and ADHD Twin Study in Sweden (RATSS). *Twin Res Hum Genet* 2014; **17**(3)**:** 164-176.

2. Myers L *et al.* Minor physical anomalies in neurodevelopmental disorders: a twin study. *Child Adolesc Psychiatry Ment Health* 2017; **11:** 57.

3. Wincent J *et al.* Copy Number Variations in Children with Brain Malformations and Refractory Epilepsy. *American Journal of Medical Genetics Part A* 2015; **167**(3)**:** 512-523.

4. Stamouli S *et al.* Copy Number Variation Analysis of 100 Twin Pairs Enriched for Neurodevelopmental Disorders. *Twin research and human genetics : the official journal of the International Society for Twin Studies* 2018; **21**(1)**:** 1-11.

5. Tammimies K *et al.* Molecular Diagnostic Yield of Chromosomal Microarray Analysis and Whole-Exome Sequencing in Children With Autism Spectrum Disorder. *Jama* 2015; **314**(9)**:** 895-903.

6. Pinto D *et al.* Convergence of genes and cellular pathways dysregulated in autism spectrum disorders. *American journal of human genetics* 2014; **94**(5)**:** 677-694.

7. Kohler S *et al.* The Human Phenotype Ontology project: linking molecular biology and disease through phenotype data. *Nucleic Acids Res* 2014; **42**(D1)**:** D966-D974.

8. De Rubeis S *et al.* Synaptic, transcriptional and chromatin genes disrupted in autism. *Nature* 2014; **515**(7526)**:** 209-U119.

9. Iossifov I *et al.* The contribution of de novo coding mutations to autism spectrum disorder. *Nature* 2014; **515**(7526)**:** 216-U136.

10. Richards S *et al.* Standards and guidelines for the interpretation of sequence variants: a joint consensus recommendation of the American College of Medical Genetics and Genomics and the Association for Molecular Pathology. *Genetics in Medicine* 2015; **17**(5)**:** 405-424.

11. Uhlin E *et al.* Derivation of human iPS cell lines from monozygotic twins in defined and xeno free conditions. *Stem cell research* 2017; **18:** 22-25.

12. Muller FJ *et al.* A bioinformatic assay for pluripotency in human cells. *Nat Methods* 2011; **8**(4)**:** 315-U354.

13. Chambers SM *et al.* Highly efficient neural conversion of human ES and iPS cells by dual inhibition of SMAD signaling. *Nat Biotechnol* 2009; **27**(3)**:** 275-280.

14. Dzyubenko E, Rozenberg A, Hermann DM, Faissner A. Colocalization of synapse marker proteins evaluated by STED-microscopy reveals patterns of neuronal synapse distribution in vitro. *J Neurosci Methods* 2016; **273:** 149-159.

15. Picelli S *et al.* Smart-seq2 for sensitive full-length transcriptome profiling in single cells. *Nat Methods* 2013; **10**(11)**:** 1096-1098.

16. Li WV, Li JJ. An accurate and robust imputation method scImpute for single-cell RNA-seq data. *Nature communications* 2018; **9**(1)**:** 997.

17. Butler A, Hoffman P, Smibert P, Papalexi E, Satija R. Integrating single-cell transcriptomic data across different conditions, technologies, and species. *Nat Biotechnol* 2018; **36**(5)**:** 411-420.

18. Sanders SJ *et al.* Insights into Autism Spectrum Disorder Genomic Architecture and Biology from 71 Risk Loci. *Neuron* 2015; **87**(6)**:** 1215-1233.

19. Yuen RKC *et al.* Whole genome sequencing resource identifies 18 new candidate genes for autism spectrum disorder. *Nat Neurosci* 2017; **20**(4)**:** 602-+.

20. Satterstrom FK *et al.* Large-scale exome sequencing study implicates both developmental and functional changes in the neurobiology of autism. *bioRxiv* 2019**:** 484113.

21. Coe BP *et al.* Neurodevelopmental disease genes implicated by de novo mutation and copy number variation morbidity. *Nat Genet* 2019; **51**(1)**:** 106-116.

22. Subramanian A *et al.* Gene set enrichment analysis: a knowledge-based approach for interpreting genome-wide expression profiles. *Proc Natl Acad Sci U S A* 2005; **102**(43)**:** 15545-15550.

23. Reimand J *et al.* Pathway enrichment analysis and visualization of omics data using g:Profiler, GSEA, Cytoscape and EnrichmentMap. *Nature protocols* 2019; **14**(2)**:** 482-517.

24. Shannon P *et al.* Cytoscape: A software environment for integrated models of biomolecular interaction networks. *Genome Res* 2003; **13**(11)**:** 2498-2504.

25. Merico D, Isserlin R, Stueker O, Emili A, Bader GD. Enrichment Map: A Network-Based Method for Gene-Set Enrichment Visualization and Interpretation. *PloS one* 2010; **5**(11).

26. Baron M *et al.* A Single-Cell Transcriptomic Map of the Human and Mouse Pancreas Reveals Inter- and Intra-cell Population Structure. *Cell Syst* 2016; **3**(4)**:** 346-360 e344.

27. Pachitariu M, Stringer C, Harris KD. Robustness of Spike Deconvolution for Neuronal Calcium Imaging. *The Journal of neuroscience : the official journal of the Society for Neuroscience* 2018; **38**(37)**:** 7976-7985.

28. Forsberg D *et al.* Functional Stem Cell Integration into Neural Networks Assessed by Organotypic Slice Cultures. *Curr Protoc Stem Cell Biol* 2017; **42:** 2D 13 11-12D 13 30.

29. Edelstein AD *et al.* Advanced methods of microscope control using muManager software. *J Biol Methods* 2014; **1**(2).

30. Jia H, Rochefort NL, Chen X, Konnerth A. In vivo two-photon imaging of sensory-evoked dendritic calcium signals in cortical neurons. *Nature protocols* 2011; **6**(1)**:** 28-35.

31. Artimovich E, Jackson RK, Kilander MBC, Lin YC, Nestor MW. PeakCaller: an automated graphical interface for the quantification of intracellular calcium obtained by high-content screening. *Bmc Neurosci* 2017; **18**.

32. Isaksson J *et al.* EU-AIMS Longitudinal European Autism Project (LEAP): the autism twin cohort. *Molecular autism* 2018; **9:** 26.

33. Edden RAE, Puts NAJ, Harris AD, Barker PB, Evans CJ. Gannet: A Batch-Processing Tool for the Quantitative Analysis of Gamma-Aminobutyric Acid-Edited MR Spectroscopy Spectra. *J Magn Reson Imaging* 2014; **40**(6)**:** 1445-1452.

34. Harris AD, Puts NAJ, Edden RAE. Tissue correction for GABA-edited MRS: Considerations of voxel composition, tissue segmentation, and tissue relaxations. *J Magn Reson Imaging* 2015; **42**(5)**:** 1431-1440.

## Supplemental Tables and Figures

### Supplementary Table 1: Psychometric profile of $ASD_{CASK\_SS}$ case and co-twin

| | $ASD_{CASK\_SS}$ case | Co-Twin |
|---|---|---|
| Early development | Language: Normal<br>Motoric: Normal<br>Toilet training: day-time enuresis until age 6y<br>First noted abnormalities: 4-5y | Language: Normal<br>Motoric: Normal<br>Toilet training: Normal<br>First noted abnormalities:9y |
| Somatic | 1y: Colic and regurgitations<br>Eczema<br>From 2 years: Sleeping disorder –Insomnia<br>5y: Slight hearing impairment - right ear<br>From 5y: Visual impairment (hyperopia); prescription for glasses | 1y: Colic<br>Allergies<br><br>From 5y: Visual impairment (hyperopia; prescription for glasses |
| Psychiatric diagnoses (DSM-IV) from Child and Youth Psychiatry | ADHD, Predominantly Inattentive Type (314.00)<br>PDD NOS (299.80) | ADHD, Predominantly Inattentive Type (314.00)<br>PDD NOS (299.80) |
| Intellectual level | GAI: 121, VCI: 106, PCI: 129 | GAI: 121, VCI: 110, PCI:125 |
| Adaptive functioning | GAF: 87 | GAF: 82 |
| ADHD deficits | Global Index: 7; AN: 7; AH: 7 | Global Index: 7; AN: 11; AH: 4 |
| Autism symptoms | AQ: 73<br>SRS total score: 69<br>ADI-R: 11-7-3-0<br>ADOS total: 2-4-1-0 | AQ: 60<br>SRS total score: 53<br>ADI-R: 11-7-0-0<br>ADOS total: 2-3-2-0 |
| Executive functioning | The Tower total scale score: 10<br>WCST total errors t-score: 61 | The Tower total scale score: 11<br>WCST total errors t-score: 65 |
| Psychiatric symptoms | Insomnia, Tics, Anxiety | - |
| Medications at time for assessment | Concerta, Strattera | No medication |
| Morphological features | Hypermobility in fingers<br>Long eyelashes<br>Hypopigmentation (i.e., vitiligo)<br>Inverted mammillary glands<br>Abnormal tear production-tears easily<br>Vision impairment<br>Overbite (teeth)<br>Sloping shoulders<br>Light hair | Overweight<br>Hitchhiker thumb<br>Long eyelashes<br>Vision impairment<br>Hypertelorism<br>Long palpebral fissure<br>Abnormal tear production-tears easily<br>High palate<br>Overbite (teeth)<br>Small ears<br>Preauricular skin tag<br>Light hair<br>Sandal gap |

**Supplementary Table 2: Bulk-Seq CASK mutant isoform detection**

| **CellLine** | **ASD_CASK_SS** | | | | | **Male Control** | | | | | **Female Control** | | | | | **MICPCH_CASK_dup4/5** | | | | |
|---|---|---|---|---|---|---|---|---|---|---|---|---|---|---|---|---|---|---|---|---|
| **Replicate** | 1 | 2 | 3 | 4 | 5 | 1 | 2 | 3 | 4 | 5 | 1 | 2 | 3 | 4 | 5 | 1 | 2 | 3 | 4 | 5 |
| **WT reads (normal splice)** | 102 | 60 | 61 | 53 | 71 | 146 | 135 | 126 | 191 | 114 | 156 | 232 | 117 | 163 | 141 | 178 | 190 | 177 | 137 | 143 |
| **14+ reads (T nucleotide)** | 3 | 2 | 0 | 1 | 3 | 0 | 0 | 0 | 0 | 0 | 0 | 0 | 0 | 0 | 0 | 0 | 0 | 0 | 0 | 0 |
| **14+ reads (G nucleotide)** | 1 | 3 | 2 | 0 | 0 | 0 | 2 | 1 | 0 | 2 | 0 | 0 | 0 | 0 | 0 | 0 | 1 | 0 | 0 | 0 |
| **Delta14 reads(skipg exon)** | 7 | 8 | 0 | 8 | 2 | 0 | 0 | 0 | 0 | 0 | 0 | 0 | 0 | 0 | 0 | 0 | 0 | 0 | 0 | 0 |
| **WT reads (exon 4-5 junction)** | | | | | | | | | | | 180 | 203 | 136 | 171 | 118 | 190 | 238 | 216 | 176 | 168 |
| **Dup4/5 reads (exon 5-4 junction)** | ND | ND | ND | ND | ND | ND | ND | ND | ND | ND | 1 | 0 | 1 | 0 | 1 | 30 | 25 | 32 | 21 | 32 |
| **Delta14/WT** | 0.07 | 0.13 | 0.00 | 0.15 | 0.03 | 0.00 | 0.00 | 0.00 | 0.00 | 0.00 | 0.00 | 0.00 | 0.00 | 0.00 | 0.00 | 0.00 | 0.00 | 0.00 | 0.00 | 0.00 |
| **14+/WT** | 0.04 | 0.08 | 0.03 | 0.02 | 0.04 | 0.00 | 0.01 | 0.01 | 0.00 | 0.02 | 0.00 | 0.00 | 0.00 | 0.00 | 0.00 | 0.00 | 0.01 | 0.00 | 0.00 | 0.00 |
| **Dup4/5/WT** | ND | ND | ND | ND | ND | ND | ND | ND | ND | ND | 0.01 | 0.00 | 0.01 | 0.00 | 0.01 | 0.16 | 0.11 | 0.15 | 0.12 | 0.19 |
| **avg Delta14** | 0.08 | | | | | 0.00 | | | | | 0.00 | | | | | 0.00 | | | | |
| **avg 14+** | 0.04 | | | | | 0.01 | | | | | 0.00 | | | | | 0.00 | | | | |
| **avg Dup4/5** | ND | | | | | ND | | | | | 0.00 | | | | | 0.14 | | | | |
| **stdev Delta14** | 0.06 | | | | | 0.00 | | | | | 0.00 | | | | | 0.00 | | | | |
| **stdev14+** | 0.02 | | | | | 0.01 | | | | | 0.00 | | | | | 0.00 | | | | |
| **stdev Dup4/5** | ND | | | | | ND | | | | | 0.00 | | | | | 0.03 | | | | |
| | | | | ND = Not determined | | | | | | | | | | | | | | | | |

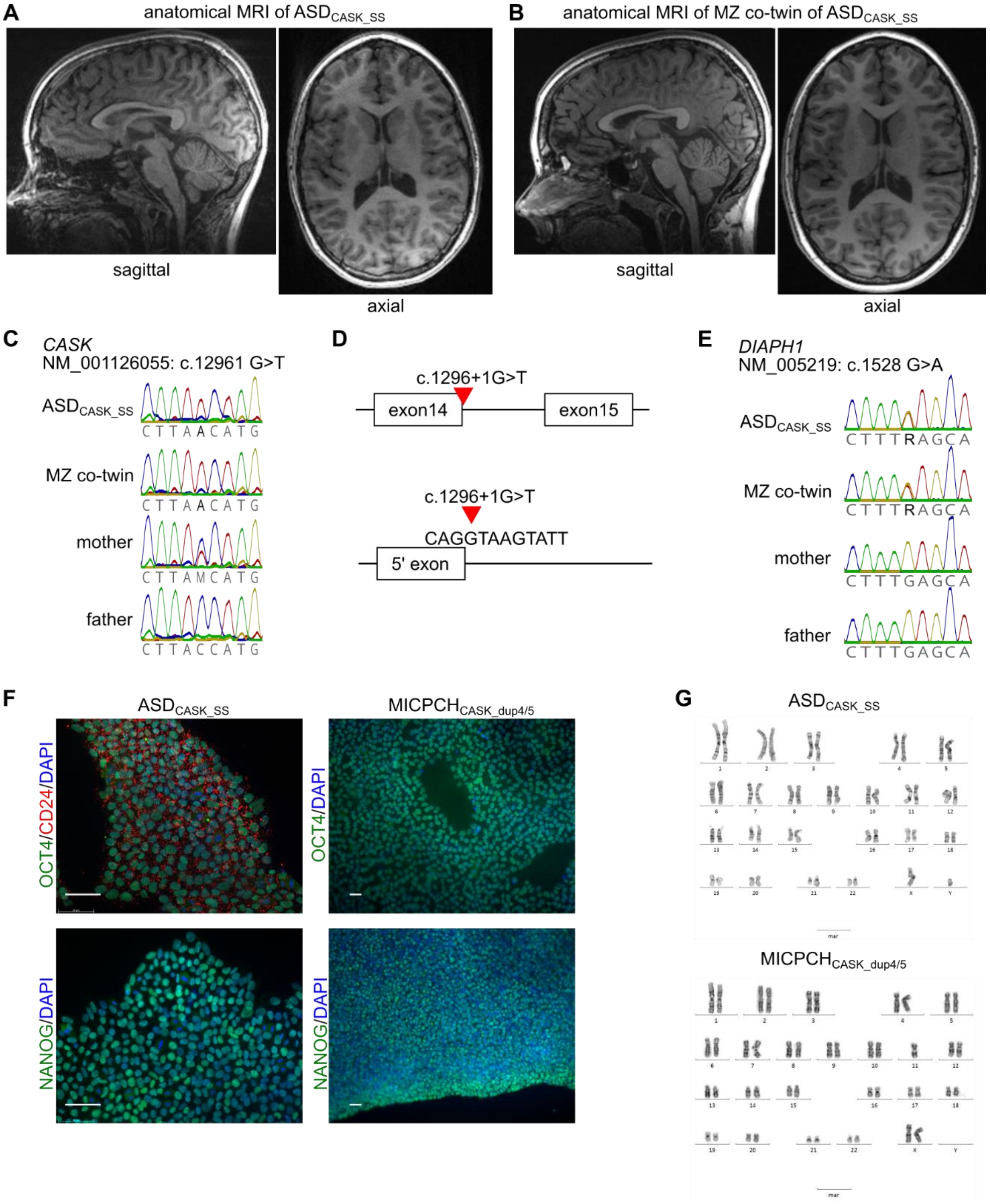


**Figure S1.** Sagittal and axial anatomical magnetic resonance images of **a** ASD$_{CASK_SS}$ and **b** his monozygotic co-twin. **c** Sanger sequencing validation of the CASK variant NM_001126055: c.1296+1 G>T detected in whole exome sequencing. Sequencing was performed on genomic DNA obtained from blood of individual ASD$_{CASK_SS}$, his co-twin and parents. **d** Schematic location of the CASK splice site variant between exon 14 and intron 14. The mutation changes the +1 guanine (G) to a thymine (T), which is not represented in the position weight matrix of donor splice sites. **e** Sanger sequencing validation of the DIAPH1 variant NM_005219: c.1528 G>A detected in whole

exome sequencing. Sequencing was performed on genomic DNA obtained from blood of individual $ASD_{CASK\_SS}$, his co-twin and parents. **f** Pluripotency marker NANOG and OCT4 staining in iPSC cells derived from individuals $ASD_{CASK\_SS}$ and $MICPCH_{CASK\_dup4/5}$. Scale = 50 µm. **g** Karyotypes of iPSC clones obtained from $ASD_{CASK\_SS}$ and $MICPCH_{CASK\_dup\_4/5}$.

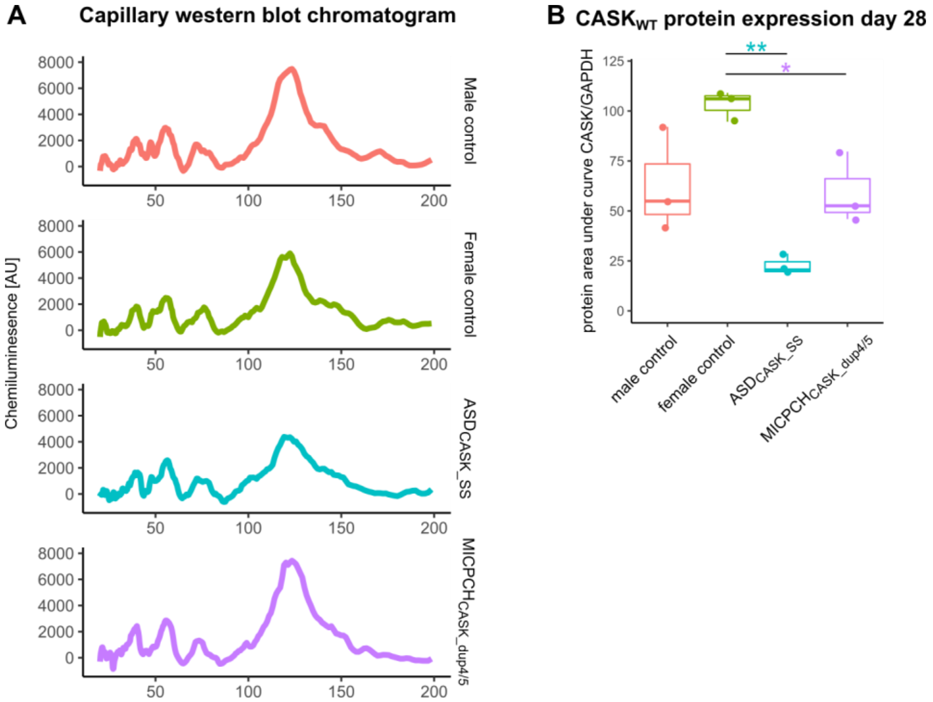


**Figure S2. a** Chromatogram of protein samples stained with CASK antibody and run on capillary western blot. **b** CASK mRNA and protein expression at 28 days of neuronal differentiation. Statistical difference was calculated with ANOVA and post-hoc Tukey. *<0.05, **<0.01, ***<0.001.

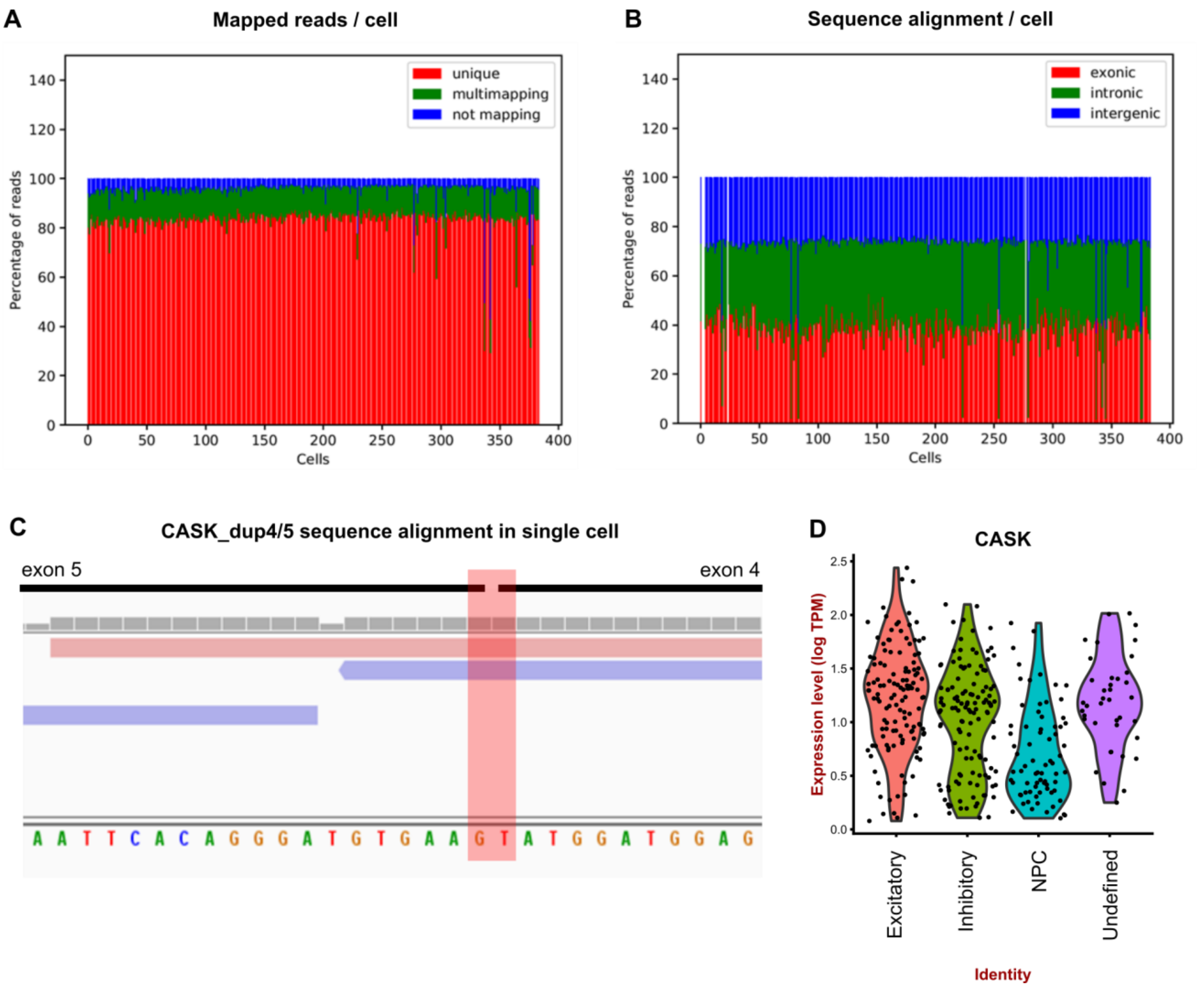


**Figure S3. a** Quality check of SMART-Seq2 data showing consistent uniquely mapped reads per cell and **b** comparable mapping to exonic, intronic and intergenic regions. **c** Detection of $CASK_{dup4\_5}$ variant in SMART-Seq2 sequencing of one cell shown in IGV browser. In this cell, two reads align to the unique exon5 to exon 4 junction of the $MICPCH_{CASK\_dup4/5}$ case. **d** Violin plot of CASK expression in all sequenced cells grouped into identified cell-types.

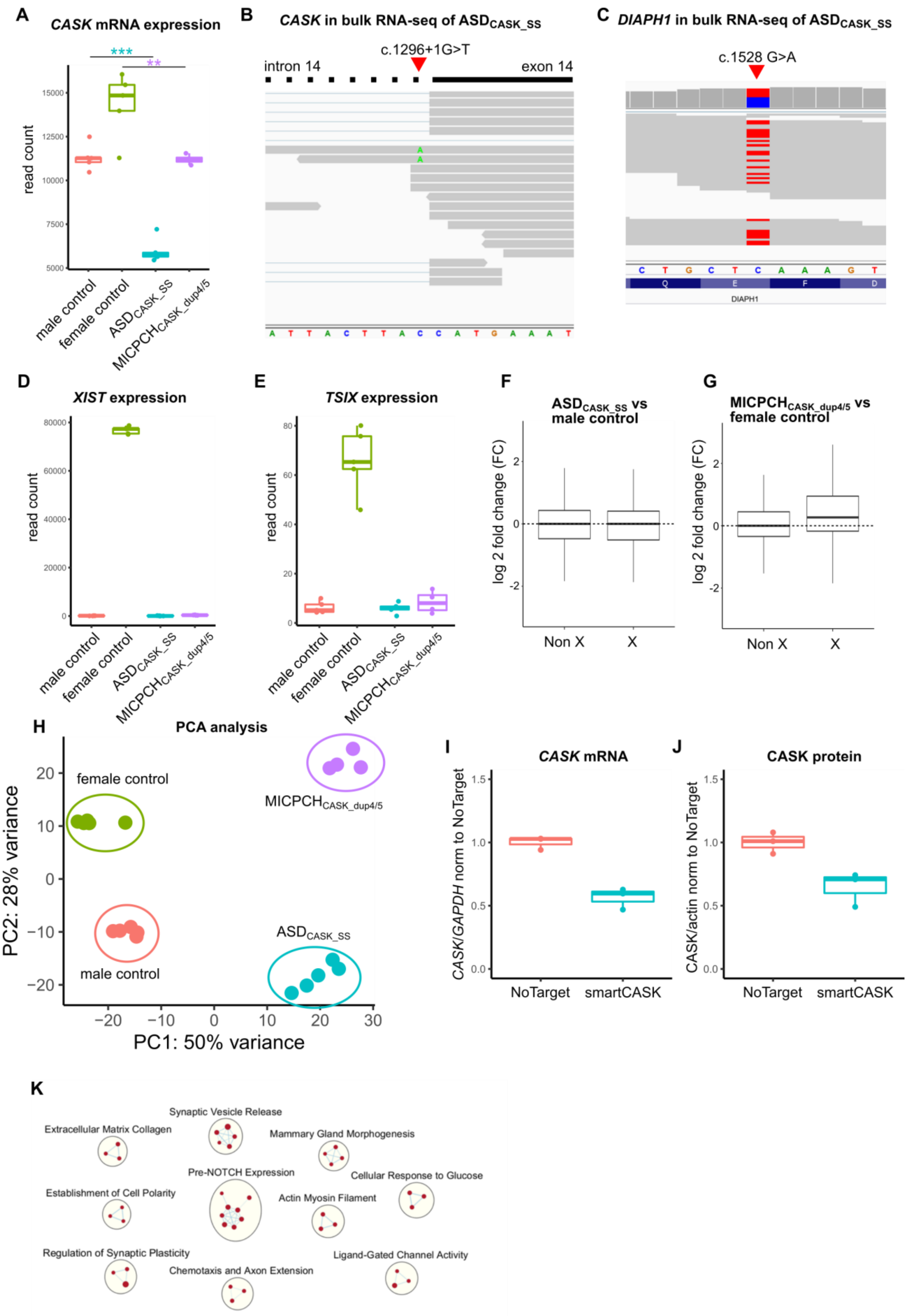
A
CASK mRNA expression
read count
male control
female control
ASD CASK_SS
MICPCH CASK_dup4/5
B
CASK in bulk RNA-seq of ASD CASK_SS
c.1296+1G>T
intron 14
exon 14
C
DIAPH1 in bulk RNA-seq of ASD CASK_SS
c.1528 G>A
DIAPH1
D
XIST expression
E
TSIX expression
F
ASD CASK_SS vs male control
log 2 fold change (FC)
Non X
X
G
MICPCH CASK_dup4/5 vs female control
H
PCA analysis
female control
male control
MICPCH CASK_dup4/5
ASD CASK_SS
PC1: 50% variance
PC2: 28% variance
I
CASK mRNA
CASK/GAPDH norm to NoTarget
NoTarget
smartCASK
J
CASK protein
CASK/actin norm to NoTarget
K
Synaptic Vesicle Release
Extracellular Matrix Collagen
Mammary Gland Morphogenesis
Pre-NOTCH Expression
Cellular Response to Glucose
Establishment of Cell Polarity
Actin Myosin Filament
Regulation of Synaptic Plasticity
Ligand-Gated Channel Activity
Chemotaxis and Axon Extension

**Figure S4. a** CASK mRNA expression in bulk RNA samples of cases and controls validates qPCR expression results. **b** Detection of RNA Sequence reads at the exon14-intron14 boundary of one $ASD_{CASK\_SS}$ sample. Sequence reads provide evidence for $CASK_{WT}$ and $CASK_{14+}$ with reference G and mutated T nucleotide. **c** Detection of *de novo* DIAPH1 variant in RNA sequence reads of $ASD_{CASK\_SS}$ showing biallelic expression of wildtype and mutant allele. **d,e** Expression of *XIST* and *TSIX* non-coding RNAs in cases and controls showing high expression only in the female control cell line. **f,g** Expression of non-X-chromosome and X-chromosome genes between **f** $ASD_{CASK\_SS}$ and male control and **g** $MICPCH_{CASK\_dup4/5}$ and female control. **h** PCA of bulk RNA-sequence samples indicating separation of cases and control along PC1 and males and females along PC2. **i,j** Expression of CASK mRNA and protein after siRNA mediated knockdown indicating 60-70% remaining CASK expression in comparison to a no-target siRNA control. **k** GSEA enriched pathways of upregulated genes after siRNA knockdown.

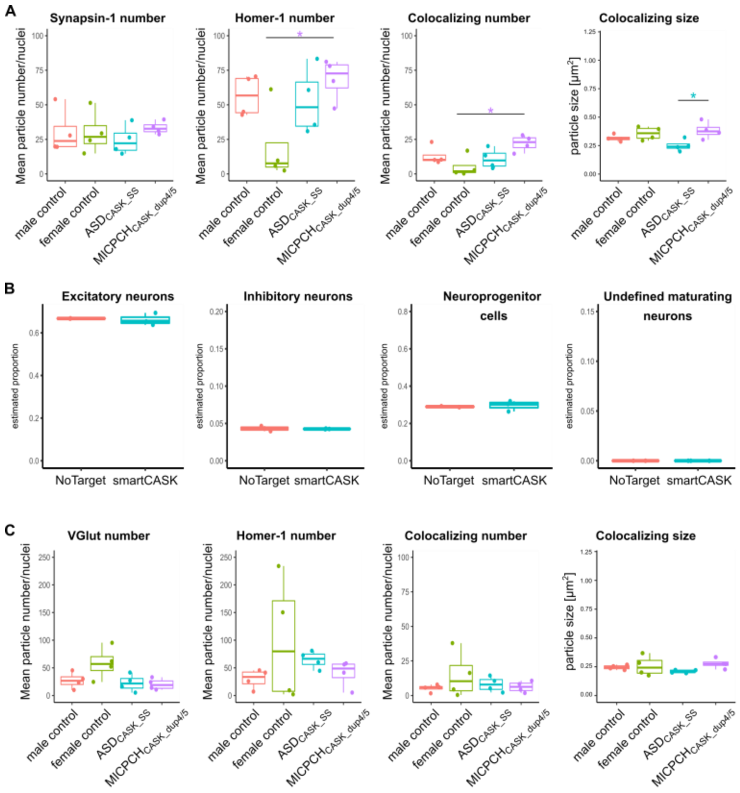


**Figure S5. a** Synapse Counter results for Synapsin-1/Homer-1 co-staining of case and control cell lines differentiated for 28 days. The panel of graphs shows the Synapsin-1 and Homer-1 number per nuclei, the number of co-localizing region and the mean size of co-localizing sizes per biological sample. Number of replicates and statistical analysis as stated in legend of Figure 5. **b** Deconvolution of cell populations in siRNA knockdown RNA sequencing samples from MICPCH$_{CASK_dup4/5}$ single-cell RNA-sequencing dataset. **c** Synapse Counter results for VGlut/Homer-1 co-staining of case and control cell lines differentiated for 28 days. The panel of graphs shows the VGlut and Homer-1 number per nuclei, the number of co-localizing region and the mean size of co-localizing sizes per biological sample (n=4).

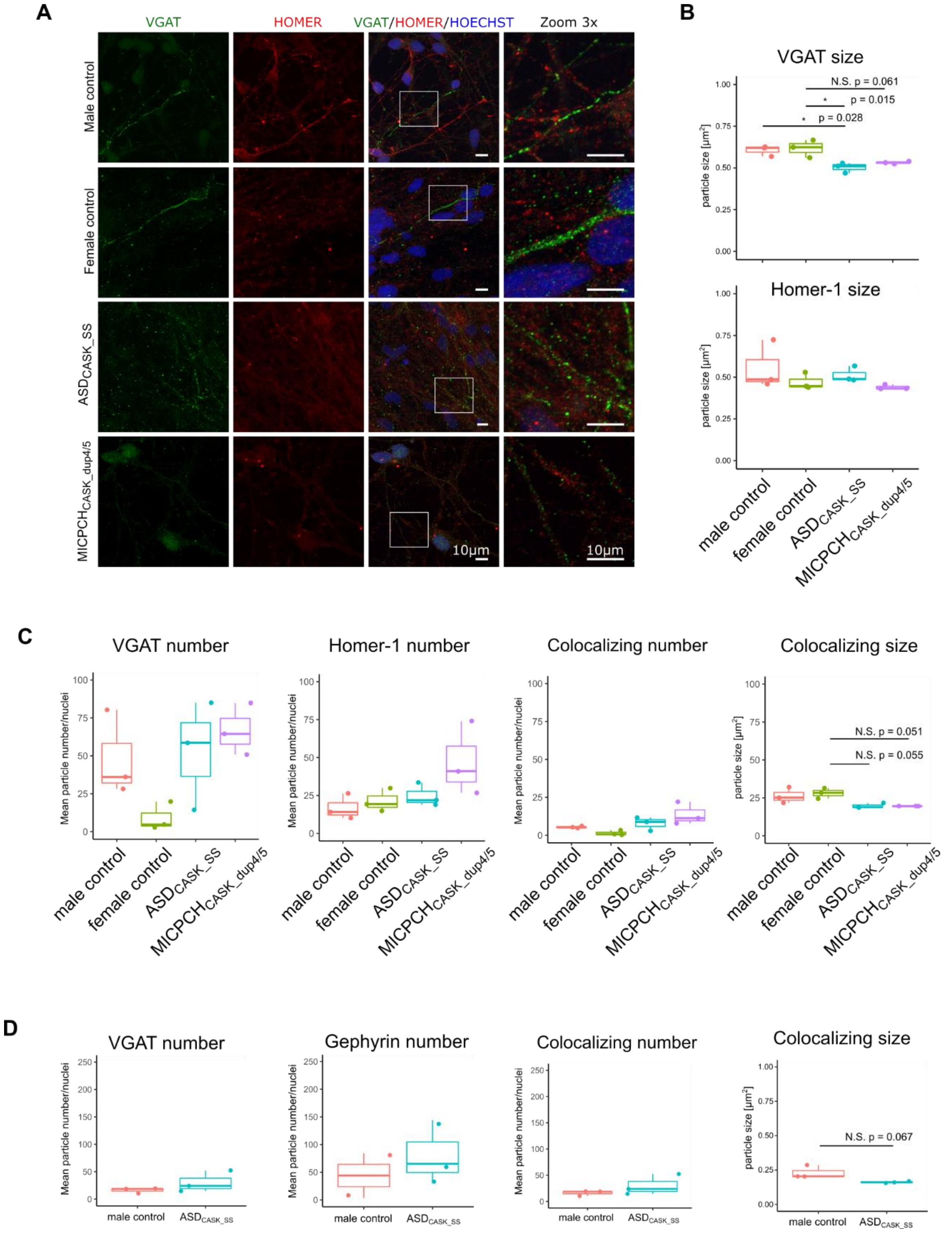


**Figure S6. a** Representative confocal microscopy images of neurons differentiated for 28 days and immunostained for inhibitory VGAT (green), Homer-1 (red) and Hoechst (blue). Scale = 10µm. **b** Quantification of VGAT and Homer-1 particle size (n=3 per cell line). Statistical differences between cell lines were calculated using ANOVA with post-hoc Tukey HSD. *p < 0.05, **p < 0.01, ***p < 0.001. **c** Synapse Counter results for VGAT/Homer-1 co-staining of case and control

cell lines differentiated for 28 days. The panel of graphs shows the VGAT and Homer-1 number per nuclei, the number of co-localizing region and the mean size of co-localizing sizes per biological sample (n=3). **d** Synapse Counter results for VGAT/gephyrin co-staining of $ASD_{CASK\_SS}$ and male control cell lines differentiated for 28 days. The panel of graphs shows the VGAT and gephyrin number per nuclei, the number of co-localizing region and the mean size of co-localizing sizes per biological sample (n=3).

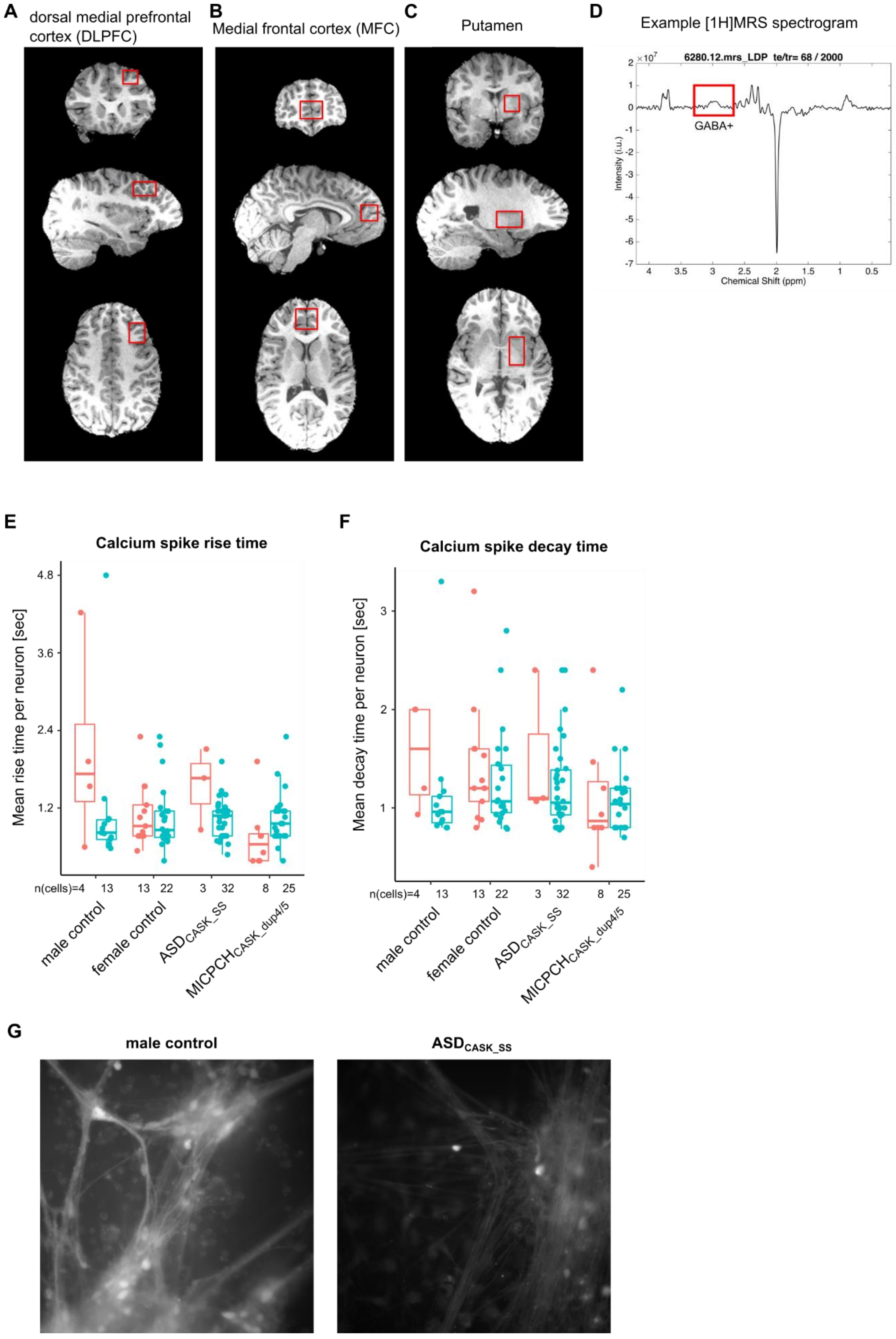
A
dorsal medial prefrontal cortex (DLPFC)
B
Medial frontal cortex (MFC)
C
Putamen
D
Example [1H]MRS spectrogram
6280.12.mrs_LDP te/tr= 68 / 2000
GABA+
Intensity (i.u.)
Chemical Shift (ppm)
E
Calcium spike rise time
Mean rise time per neuron [sec]
n(cells)=4 13 13 22 3 32 8 25
male control
female control
ASDCASK_SS
MICPCHCASK_dup4/5
F
Calcium spike decay time
Mean decay time per neuron [sec]
n(cells)=4 13 13 22 3 32 8 25
male control
female control
ASDCASK_SS
MICPCHCASK_dup4/5
G
male control
ASDCASK_SS

**Figure S7. a-c** Example MR images underlying [1H]MRS, indicating the voxel regions used to detect GABA concentrations in **a** the DLPFC, **b** MFC and **c** putamen. **d** Example [1H]MRS spectrum obtained from a 13.6 ml voxel located at the DLPFC, indicating the spectral peak of GABA. **e** Calcium spike rise and **f** decay times in active neurons of mutation carriers and controls differentiated for 4 and 5 weeks. Number of total neurons below x-axis. Biological replicates at 4-weeks: Female control (n=2), male control (n=2), $ASD_{CASK\_SS}$ (n=1) and $MICPCH_{CASK\_dup4/5}$ (n=2). 5-weeks: Female control (n=2), male control (n=2), $ASD_{CASK\_SS}$ (n=5) and $MICPCH_{CASK\_dup4/5}$ (n=2). Statistical differences between cell lines were calculated using pairwise Wilcoxon Rank Sum test followed by Bonferroni correction. **g** Representative images of Fluo-8 staining in 4-week differentiated male control and $ASD_{CASK\_SS}$ neuronal cultures.
